# PoLambRimetry: a multispectral polarimetric atlas of lamb brain

**DOI:** 10.1117/1.JBO.29.9.096002

**Published:** 2024-09-17

**Authors:** Verónica Mieites, Giulio Anichini, Ji Qi, Kevin O’Neill, Olga M. Conde, Daniel S. Elson

**Affiliations:** a University of Cantabria, Photonics Engineering Group, Santander, Spain; b Valdecilla Health Research Institute (IDIVAL), Santander, Spain; c Imperial College London, Department of Brain Sciences, London, United Kingdom; d Research Centre for Fundamental Research, Zhejiang Lab, Hangzhou, China; e Bioengineering, Biomaterials and Nanomedicine Research Network (CIBER-BBN), Madrid, Spain; f Imperial College London, Hamlyn Centre for Robotic Surgery, Department of Surgery and Cancer, London, United Kingdom

**Keywords:** Mueller matrix imaging, multispectral imaging, wide field, non-invasive imaging, brain tissue, optical properties

## Abstract

**Significance:**

Mueller matrix imaging (MMI) is a comprehensive form of polarization imaging useful for assessing structural changes. However, there is limited literature on the polarimetric properties of brain specimens, especially with multispectral analysis.

**Aim:**

We aim to employ multispectral MMI for an exhaustive polarimetric analysis of brain structures, providing a reference dataset for future studies and enhancing the understanding of brain anatomy for clinicians and researchers.

**Approach:**

A multispectral wide-field MMI system was used to measure six fresh lamb brain specimens. Multiple decomposition methods (forward polar, symmetric, and differential) and polarization invariants (indices of polarimetric purity and anisotropy coefficients) have been calculated to obtain a complete polarimetric description of the samples. A total of 16 labels based on major brain structures, including grey matter (GM) and white matter (WM), were identified. K-nearest neighbors classification was used to distinguish between GM and WM and validate the feasibility of MMI for WM identification.

**Results:**

As the wavelength increases, both depolarization and retardance increase, suggesting enhanced tissue penetration into deeper layers. Moreover, utilizing multiple wavelengths allowed us to track dynamic shifts in the optical axis of retardance within the brain tissue, providing insights into morphological changes in WM beneath the cortical surface. The use of multispectral data for classification outperformed all results obtained with single-wavelength data and provided over 95% accuracy for the test dataset.

**Conclusions:**

The consistency of these observations highlights the potential of multispectral wide-field MMI as a non-invasive and effective technique for investigating the brain’s architecture.

## 1 Introduction

In the landscape of medicine, clinicians rely on imaging modalities for precise diagnostics and surgical interventions. Among the diverse array of imaging techniques, polarization imaging stands out as a potential tool capable of discerning structural changes through tissue anisotropy.^1^ The polarizing properties of a sample are affected by absorption, scattering, and overall structure, which will vary depending on the tissue type. Moreover, the wavelength dependency of the optical parameters^2^ adds another layer of complexity to imaging processes. The choice of wavelength and bandwidth not only influences the resolution of the obtained images but also determines the optical penetration depth into the biological sample.

The most comprehensive form of polarization imaging is achieved through Mueller matrix imaging (MMI), which enables the acquisition of all inherent polarization properties in a given sample.^3^ In medicine, MMI-based diagnosis finds diverse applications in cancer detection, particularly in head and neck,^4^ breast,^5^^–^^7^ colon,^8^^–^^11^ lung,^12^ skin,^13^^–^^15^ cervix,^16^^–^^18^ and liver.^18^^,^^19^ Beyond cancer applications, MMI was used for assessing fiber structures in soft tissue membranes,^20^ articular cartilage,^21^ and skin burns.^22^ These applications collectively validate MMI as a suitable technique to assess anisotropy changes in samples, which reflect the structural alterations that lead to polarization variations.

Brain imaging, the primary focus of this work, has been explored in several studies through MMI. Depolarization, retardance, and diattenuation emerge as the predominantly examined polarization properties, alongside the Mueller matrices themselves. Specifically, MMI applied to the brain has revealed the correlation between albedo and depolarization,^23^ improved the visualization of the distribution and orientation of white matter tracts,^24^^–^^27^ investigated the progression of Alzheimer’s,^28^ and distinguished between healthy brain tissue and glioblastoma.^29^

As illustrated in Table 1, the field of brain tissue polarimetry has seen a diverse range of experimental approaches, including various sample types (human and animal, fresh and fixed, and bulk and sliced), imaging systems (transmission, reflection, and snapshot), wavelengths, and decomposition methods. Human samples are typically analyzed in a fixed and sliced state due to the challenges associated with *in vivo* imaging. By contrast, a variety of animal models, both fresh and fixed, have been used to explore brain polarimetric properties under different experimental conditions. Reflectance systems, enabling the analysis of thicker and bulk samples, have emerged as a dominant imaging modality, likely due to their closer resemblance and applicability in real-world scenarios, although transmission setups are useful for histological analysis. Multiple decomposition methods have been applied to the Mueller matrix analysis of brain samples, each offering unique insights into the underlying polarization properties of tissues. The forward polar or Lu–Chipman decomposition has arisen as a prevalent tool in brain imaging, owing to its simplicity, interpretability, and established utility. However, other methods, such as the differential and arrow decompositions, have also been proven valuable in differentiating between tumoral tissue and various brain structures, including grey and white matter. Although wavelengths centered around 470, 550, and 645 nm are commonly employed in brain studies, likely due to their correspondence with RGB color bands in regular cameras, multi-wavelength investigations remain limited. The heterogeneity of sample types in Mueller imaging studies of brain tissue, coupled with the variation in experimental conditions and the limited exploration of multi-wavelength effects, poses challenges for extrapolating findings across different wavelengths and samples. This complicates the task of identifying the optimal wavelength for a given study and underscores the need for further investigation into the spectral and tissue-specific polarimetric properties of brain tissue.

**Table 1:** Selected references that apply Mueller matrix imaging for brain analysis.

Brain samples	System	Wavelength of reported results (nm)	Method	Ref.
Pig, fresh, slices (2 mm, 1 cm)	Rx/Tx	635	FPD	23
Human, fixed, slices (60 μm)	Tx	632.8	DD	30
Human, fixed, sections (1 cm); Cow, fresh, sections (1 cm)	Rx	550, 600, and 650	FPD	24
Mouse, fixed, slices ( μm)	Rx	450, 500, and 550	TD	28
Human, fixed, slices	Rx (microscope)	700	BSV	31
Cow, fresh, sections and bulk	Rx	550 and 650	FPD	25
Goat, fresh, sections (1.5 cm); Cow, fresh, sections (1.5 cm)	Rx-SFDI	630	FPD	32
Human, fixed, slices (6 μm)	Rx (3 × 3)	630	FPD MMT	29
Ferret, fixed, bulk	Rx (microscope)	405, 473, 543, and 632	FPD	33
Mouse, fixed, slices (10 μm, 500 μm)	PA - Rx	527	System specific	34
Pig, fresh, sections and bulk	Rx	550 and 650	FPD	26
Cow, fresh, section	Rx	470	AD	35
Human, fixed, slices (5 and 10 μm)	Rx (microscope)	550	FPD DD	27
Pig, fresh and fixed, sections	Rx	550	FPD	36
Cow, fresh, sections (~cm)	Rx	470	AD	37
Cow, fresh, sections (~cm)	Rx	470	FPD, PPI	38
Pig, fresh, sections (3 cm)	Rx	550	FPD	39
Human, fresh, sections	Rx	550	FPD	40
Ferret, fixed, bulk	Rx (microscope)	405, 442, 473, 545, 605, and 632	FPD	41
Pig, fresh, sections (3 cm)	Rx	550	FPD	42

“System” column: Rx and Tx stand for reflection and transmission, respectively, SFDI, spatial frequency domain imaging; PA, photoacoustic. 3×3 indicates that the system is not a complete Mueller polarimeter. “Method” column: FPD, forward polar decomposition; DD, differential decomposition; TD, total diattenuation; BSV, backscattered stokes vector; MMT, Mueller matrix transformation; AD, arrow decomposition.

Despite the strides made in applying MMI to the brain, to the authors’ knowledge, there is no bibliographic reference that focuses on reporting with detail the polarimetric properties of healthy whole brain specimens and their main sub-structures. Building upon our preliminary findings,^43^ we propose the use of multispectral MMI of the brain combined with an exhaustive polarimetric analysis of its multiple structures. The presented methodology and experiments aim to create a dataset of Mueller matrix images, serving as a reference for future polarization studies and providing clinicians and researchers with an additional tool for the understanding of brain anatomy.

## 2 Materials and Methods

### 2.1 Sample Preparation

**Dissection**. A total of 20 regions from six specimens of locally sourced fresh lamb brain were used for this work. A summary of the acquired regions is included in Table 2. In 13 cases, a section of the specimen was performed to adequately visualize areas such as the brainstem, the corona radiata, the cerebellar white matter (arbor vitae), or the diencephalon. In the remaining seven cases, an intact lateral view of different areas (mostly cerebral or cerebellar hemispheres) was acquired. Samples were preserved at fridge temperature and dissected using a size 10 scalpel. We identified four macro-regions in our specimens: brain hemisphere (corresponding to cortex and subcortical white matter), basal ganglia/diencephalon, brainstem, and cerebellum (see Table 2).

**Table 2:** Summary of the measurements grouped by brain region.

Part	Specimens	Regions	Views
Brain hemisphere	1 to 6	11 (6 right, 5 left)	Lateral (5), medial (4), and sections (2)
Cerebellum hemisphere	2 to 4	6	Lateral (2) and medial (4)
Brainstem and pineal region	4	2	Sections (1) and medial (1)
Basal ganglia and diencephalon	5	1	Sections

**Labeling**. On the dissection images, specific white matter (WM) and grey matter (GM) regions were labeled. These were identified on the basis of their macroscopic appearances and the anatomical landmarks specific to a lamb brain. For this purpose, the specimens were compared with those available in the literature^44^ or online.^45^ The resulting labeling of different regions is showcased in Table 3.

**Table 3:** Labeled brain areas sorted by type and largest to smallest number of pixels. The values of all GM and WM areas are summarized in their respective (bold) rows.

Structure	Type	Specimens	Regions	Pixel no.
**Grey matter**	**GM**	1 to 6	20	1,369,395
Basal ganglia	GM	4 to 6	5	80,661
Superior colliculus	GM	4 to 6	5	31,073
Third ventricle	GM	6	1	9391
Inferior colliculus	GM	4, 5	3	5254
Pineal gland	GM	4	1	4302
Periaqueductal GM	GM	4	1	3326
Optic chiasm	GM	3	1	1667
Area posterema	GM	4	1	1255
Brainstem nuclei	GM	4	1	976
Claustrum	GM	5	1	611
**White matter**	**WM**	1 to 6	19	597,686
Corticospinal tracts	WM	2	1	6308
Trochlear nerve	WM	2	1	1026
Cerebellar peduncle	WM	2	1	576
**Vessels**	**Vessel**	1 to 5	6	16,104

### 2.2 System Description

**Fig. 1:**
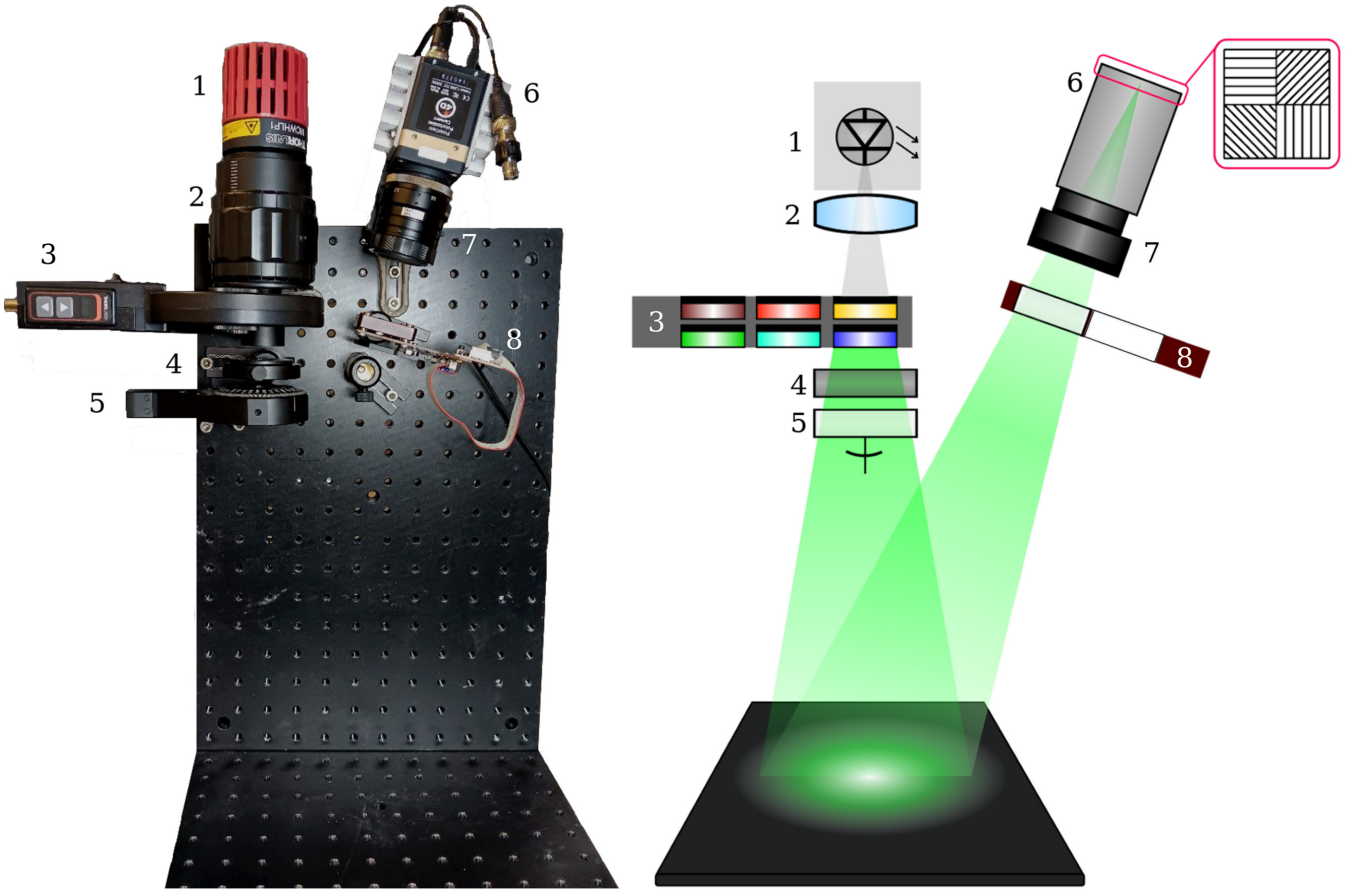
Multispectral MMI’s photograph (a) and diagram (b): (1) LED light source, (2) LED collimator, (3) filter wheel, (4) linear polarizer, (5) rotating mount with λ/4 retarding film, (6) polarization camera, (7) lens, and (8) sliding filter mount with λ/4 retarding film. The oblique detection angle is approximately 7 deg.

The imaging system utilized in this study is illustrated in Fig. 1. The setup, inspired in previously reported MMI devices,^46^^,^^47^ was arranged in reflection configuration, including a collimated white light emitting diode (LED, MCWHLP3, Thorlabs, Newton, New Jersey, United States) filtered within the wavelength range of 450 to 680 nm by six color filters in a rotating wheel (FW102C, Thorlabs). The polarization state generator (PSG) consists of a linear polarizer combined with a λ/4 retarding film housed in a motorized mount (PRM1/MZ8, Thorlabs). By rotating this mount, the device generates the four incident illumination polarization states.

In the detection branch, a polarimetric camera (PolarCam snapshot micropolarizer camera, 4D Technology, Tucson, Arizona, United States) with a wide-field lens (NMV50M1, Navitar, Rochester, New York, United States) is used as the primary detector. The camera is equipped with an array of linear polarizers positioned on top of the detection pixels at four different orientations, enabling the retrieval of all linear polarization information in a single capture. For obtaining phase information, another λ/4 retarding film is inserted in one of the two positions of the sliding filter mount (ELL6K, Thorlabs), leaving the other position empty. The camera, in conjunction with the sliding filter mount, constitutes the polarization state analyzer (PSA).

### 2.3 Measurement Procedure and Dataset Description

Six specimens of lamb brains were measured on 20 regions at six wavelengths between 450 and 680 nm (450, 500, 550, 590, 650, and 680 nm). All filters had a bandwidth of 40 nm, except for those at 590 and 680 nm, which had bandwidths of 10 nm. The exposure time was adjusted for each measurement to compensate for the light power dependency with wavelength, which resulted in exposure times between 0.3 and 3 s. The first two specimens were set on top of aluminum foil, which was later removed for the remaining specimens to minimize undesired reflections.

Each complete polarization measurement took ∼1  min and involved a total of 32 images: one for each position of the rotating retarding film (4), one for each position of the sliding filter mount (2), and one for each of the four polarizers on the camera sensor (4). This compendium of images is sufficient for deriving the Mueller matrix of the sample. Given that we used six color filters and 20 samples, a total of 3840 images were captured, which translates into 120 Mueller matrices, each with its corresponding polarization properties.

### 2.4 Polarization Properties

The Mueller matrix (M) of the sample, as constructed from the actual measured matrices ($\overset{¯}{M}$), will depend on the polarimetric response of the PSA and PSG matrices [Eq. (1)]

$$\overset{¯}{M}=\mathrm{PSA}\cdot M\cdot \mathrm{PSG}.$$ (1)

To obtain them, we used the eigenvalue calibration method (ECM), as described by Compain et al.^48^ In brief, this method is based on the measurement of known polarizing elements, also defined as calibration samples, to calculate the PSA and PSG matrices by comparing the expected matrices of the calibration samples with their actual measurements. Due to having six individual color filters, our system required a different calibration for each filter. The ECM also retrieves the error of the calibrated PSA and PSG through the study of the eigenvalues; the calibration error was, on average, 6.3%, with the highest error being 8.2% and the lowest being 4.5%.

Once obtained, the matrices M contain 4×4 elements, $M_{ij}$, i,j∈[0,3], that are typically represented in a normalized fashion, $m_{ij}=M_{ij}/M_{00}$, i,j∈[0,3], in terms of the polarizance $\vec{P}=(m_{10},m_{20},m_{30}{)}^{T}$ and diattenuation $\vec{D}=(m_{01},m_{02},m_{03}{)}^{T}$ vectors, as follows:

$$M=(\begin{array}{cccc} M_{00} & M_{01} & M_{02} & M_{03}\\ M_{10} & M_{11} & M_{12} & M_{13}\\ M_{20} & M_{21} & M_{22} & M_{23}\\ M_{30} & M_{31} & M_{32} & M_{33} \end{array})=M_{00}(\begin{array}{cccc} m_{00} & m_{01} & m_{02} & m_{03}\\ m_{10} & m_{11} & m_{12} & m_{13}\\ m_{20} & m_{21} & m_{22} & m_{23}\\ m_{30} & m_{31} & m_{32} & m_{33} \end{array})=M_{00}(\begin{array}{cc} 1 & {\overset{\to }{D}}^{T}\\ \overset{\to }{P} & m_{3\times 3} \end{array}).$$ (2)

#### 2.4.1 Verifying the validity of experimental Mueller matrices

Not all measured Mueller matrices depict a correct representation of the polarization properties of the sample. Specifically, factors such as instrumental noise, misalignments, randomization through depolarization, inhomogeneities across the penetration depth, or non-linearities such as fluorescence might lead to calculated matrices that are not physically realizable.

The ensemble criterion^49^ can be utilized to determine whether measured samples represent physically realizable Mueller matrices, which should be equivalent to a weighted average of non-depolarizing matrices. Mathematically, this implies two conditions. The first one relates to the covariance matrix of M, H(M), defined as

$$H=\frac{1}{4}\overset{3}{\underset{i,j=0}{\sum }}M_{ik}\sigma_{i}⊗\sigma_{j},$$ (3)

where $\sigma_{i}$ are the Pauli matrices and ⊗ represents the tensor product. For M to be physically realizable, all eigenvalues of H(M) must be greater than or equal to zero.^50^

The second condition of the ensemble criterion implies that the medium cannot increase the degree of polarization of the incident light. This condition is evaluated through two equations, namely, the forward and reverse passivity conditions [Eq. (4)]^51^

$$M_{00}(1+\|\overset{\to }{D}\|)\leq 1,\;M_{00}(1+\|\overset{\to }{P}\|)\leq 1.$$ (4)

#### 2.4.2 Indices of polarimetric purity indices (IPPs) and anisotropy coefficients

Mueller matrices can be characterized by their polarimetric purity, quantified by the IPPs, and their anisotropy, assessed through various anisotropy coefficients. These metrics are useful for providing insights into the depolarizing and anisotropic properties of the medium. Let $\lambda_{i}$, i∈[0,3] be the four eigenvalues of H(M), with $\lambda_{0}\geq \lambda_{1}\geq \lambda_{2}\geq \lambda_{3}\geq 0$. Let λ^i=λi/trace(H). Then, the three indices of polarimetric purity are defined as^52^^,^^53^

P1=λ^0−λ^1,P2=λ^0+λ^1−2λ^2,P3=4−λ^1, (5)

and the degree of polarimetric purity is defined as

$$P_{\Delta }=\sqrt{\frac{1}{3}(2P_{1}^{2}+\frac{2}{3}P_{2}^{2}+\frac{1}{3}P_{3}^{2})}.$$ (6)

The IPPs represent the amount of polarization maintained by the sample, taking values between 0 and 1 and satisfying the inequality $0\leq P_{1}\leq P_{2}\leq P_{3}\leq 1$.

Aside from the IPPs, the anisotropy coefficients can also be directly derived from the Mueller matrix to provide insights regarding the anisotropies present in the samples. Starting with the 3×3 sub-matrix of M, $m_{3\times 3}$, the following complementary vectors are defined:^54^

$$\overset{\to }{k}=\frac{1}{\sqrt{3}}(k_{1},k_{2},k_{3}{)}^{T},\;\overset{\to }{r}=(r_{1},r_{2},r_{3}{)}^{T},\;\overset{\to }{q}=(q_{1},q_{2},q_{3}{)}^{T},$$ (7)

where

$$m_{3\times 3}=(\begin{array}{ccc} k_{1} & r_{3} & r_{2}\\ q_{3} & k_{2} & r_{1}\\ q_{2} & q_{1} & k_{3} \end{array}).$$ (8)

By defining $\Sigma =3(1-{\left\|\overset{\to }{k}\right\|}^{2})+2{\overset{\to }{D}}^{T}\overset{\to }{P}-2{\overset{\to }{r}}^{T}\overset{\to }{q}$ and taking the individual elements of vectors $\vec{D}$, $\vec{P}$, $\vec{k}$, $\vec{r}$, and $\vec{q}$, the linear ($\alpha_{L}$) and circular ($\alpha_{C}$) anisotropy coefficients are derived as

$$\alpha_{L}=\sqrt{\frac{(D_{1}+P_{1}{)}^{2}+(r_{1}-q_{1}{)}^{2}+(D_{2}+P_{2}{)}^{2}+(r_{2}-q_{2}{)}^{2}}{\Sigma }},\;\alpha_{C}=\sqrt{\frac{(D_{3}+P_{3}{)}^{2}+(r_{3}-q_{3}{)}^{2}}{\Sigma }},$$ (9)

and the overall degree of anisotropy is derived as

$$P_{\alpha }=\sqrt{\alpha_{L}^{2}+\alpha_{C}^{2}}\leq P_{\Delta }\leq 1.$$ (10)

These coefficients are valuable for analyzing the directional dependence of the samples’ optical properties, which in turn is related to their 3D structure. However, the degree of anisotropy is inherently constrained by the degree of polarimetric purity. Highly depolarizing samples (low $P_{\Delta }$) will necessarily exhibit low anisotropy, but the converse is not always true—low anisotropy does not guarantee low polarimetric purity.

#### 2.4.3 Forward polar decomposition

Although there are many ways of decomposing M, the most commonly applied method is the forward polar (or Lu–Chipman) decomposition.^55^ The Lu–Chipman decomposition is one of the existing methods to decompose one matrix in terms of a finite series of matrices, each representing some known polarization properties. These decompositions prioritize easily interpretable results at the cost of assuming that the media can be modeled as three distinct layers ordered in one particular way. Although newer decomposition methods that do not have the same limitations have emerged, the Lu–Chipman decomposition, one of the first models in the field, continues to be the most widely applied method, particularly for analyzing Mueller matrices in brain tissue.

The forward polar decomposition is based on the assumption that any polarization property can be understood as a combination of depolarization (Δ), retardance (R), and diattenuation (D), through their respective Mueller matrices, $M_{\Delta }$, $M_{R}$, and $M_{D}$, as indicated in Eq. (11), which is typically calculated after normalizing M by its first element, $M_{00}=M(0,0)$.

$$M=M_{\Delta }\cdot M_{R}\cdot M_{D}.$$ (11)

For each decomposed Mueller matrix, their magnitudes (Δ, R, and D) are derived as follows [Eq. (12)]:

$$\Delta =1-\frac{\left|\text{trace}(M_{\Delta })-1\right|}{3}\in [0,1],\;R=\cos^{-1}(\frac{\text{trace}(M_{R})}{2}-1)\in [0,\pi ],\;D=\|\overset{\to }{D}\|=\|(M_{D}(1,0),M_{D}(2,0),M_{D}(3,0){)}^{T}\|\in [0,1].$$ (12)

The orientation of the optical axis of linear retardance of the sample (θ) is calculated by understanding the retardance as a combination of linear and circular birefringence. In this manner, θ is obtained by decomposing $M_{R}$ as a combination of a linear retarder (LR) with a retardance of δ and an optical rotation ψ [Eq. (13)]

$$M_{R}=M_{\mathrm{LR}}\cdot M_{\psi }.$$ (13)

By deriving the 16 equations inside the product of Eq. (13), the value of θ is determined as follows:^56^

$$\theta =\frac{1}{2}\;\tan^{-1}(\frac{M_{\mathrm{LR}}(3,1)-M_{\mathrm{LR}}(1,3)}{M_{\mathrm{LR}}(2,3)-M_{\mathrm{LR}}(3,2)}),$$ (14)

where the (i,j) values in $M_{\mathrm{LR}}(i,j)$ refer to the row and column of $M_{\mathrm{LR}}$, respectively. All of the derived polarization properties are wavelength-dependent as the optical properties of the sample and the penetration depth vary with the wavelength.

#### 2.4.4 Symmetric decomposition

The forward and reverse polar decompositions (the latter not discussed here) impose a specific order for the equivalent depolarizer, retarder, and diattenuator matrices. However, matrix algebra dictates that altering this order yields different equivalent element sets, each characterized by distinct matrices. Notably, the depolarizer matrix ($M_{\Delta }$) resulting from the polar decomposition can contain non-zero off-diagonal elements, implying the presence of polarizance or diattenuation, depending on the chosen matrix order. To overcome those limitations, the more recently proposed symmetric decomposition^57^ proposes a five-layer model with two retarders ($M_{R1}$ and $M_{R2}$), two diattenuators ($M_{D1}$ and $M_{D2}$), and one diagonal depolarizer ($M_{\Delta d}$) while retaining the ease of interpretability of a serial model, given as follows:

$$M=M_{D2}M_{R2}M_{\Delta d}M_{R1}^{T}M_{D1}.$$ (15)

This model, derived from the normal form of M,^58^ implies that the product matrices $M_{j2}=M_{D2}M_{R2}$ and $M_{j1}=M_{R1}^{T}M_{D1}$ are non-depolarizing (pure) components, ensuring that all depolarization is confined to $M_{\Delta d}$.

The symmetric decomposition requires the Mueller matrix M to be diagonalizable to derive the diagonal depolarizer as

$$M_{\Delta d}=(\begin{array}{cccc} d_{0} & 0 & 0 & 0\\ 0 & d_{1} & 0 & 0\\ 0 & 0 & d_{2} & 0\\ 0 & 0 & 0 & d_{3} \end{array}).$$ (16)

Specifically, given G=diag(1,−1,−1,−1), the applicability of the symmetric decomposition can be verified by checking the diagonalizability of the matrix as

$$N=GM^{T}GM.$$ (17)

To obtain the two diattenuators, vectors ${\overset{\to }{s}}_{D1}=(1,{\overset{\to }{D}}_{1}{)}^{T}$ and ${\overset{\to }{s}}_{D2}=(1,{\overset{\to }{D}}_{2}{)}^{T}$, related to the diattenuation vectors of $M_{D1}$ and $M_{D2}$, respectively, are resolved using the eigenvalue–eigenvector equations^57^

$$(M^{T}GMG){\overset{\to }{s}}_{D1}=(\overset{\to }{d_{0}}{)}^{2}{\overset{\to }{s}}_{D1},\;(MGM^{T}G){\overset{\to }{s}}_{D2}=(\overset{\to }{d_{0}}{)}^{2}{\overset{\to }{s}}_{D2}.$$ (18)

The absolute diattenuation for each $M_{Di}$ is then obtained, as indicated in Eq. (12), to verify that the inequalities $D_{1,2}\leq 1$ hold. Subsequently, applying singular value decomposition (SVD) to the intermediate matrix,

$$M^{\prime }=M_{D2}^{-1}MM_{D1}^{-1}=M_{R2}M_{\Delta d}M_{R1}^{T},$$ (19)

is enough to retrieve the remaining matrices, namely, $M_{R2}$, $M_{\Delta d}$, and $M_{R1}$.

It is worth noting that the SVD is invariant to rotations and therefore can introduce an artificial circular retardance in $M_{R1}$ and $M_{R2}$. To avoid this, the overall product

$$M_{R}=M_{R1}M_{R2}^{T},$$ (20)

which is not modified by the rotations introduced by the SVD, is used instead. The complete mathematical formulation is available in the literature.^57^^,^^59^

#### 2.4.5 Differential decomposition

In a real, macroscopic sample, considering it completely uniform along the light propagation is often not precise. Instead, the differential decomposition^60^^,^^61^ assumes that, for each infinitesimal element of the optical path length (dz), there is a differential Mueller matrix m(z) that relates to M(z) through

$$\frac{dM(z)}{dz}=m(z)M(z).$$ (21)

If m was non-dependent of z, Eq. (21) would have a solution in terms of the matrix logarithm (assuming z=1)

m=ln(M). (22)

However, it is often not the case that m is independent from z. In such cases, the matrix logarithm can still be defined as L=ln(M) and analyzed in the same way that m would be.^60^^,^^61^

Let G=diag(1,−1,−1,−1) be the Minkowski metric matrix. Then, for any matrix A, the concept of G-transpose is defined as

$$A^{G}=GA^{T}G,$$ (23)

and G-symmetry and G-antisymmetry are fulfilled when

$$A^{G}=A,\;A^{G}=-A,$$ (24)

respectively.

The matrix logarithm L is separated into its G-antisymmetric ($L_{m}$) and G-symmetric ($L_{u}$) parts as follows:

$$L=\ln(M)=L_{m}+L_{u}=\frac{1}{2}(L-GL^{T}G)+\frac{1}{2}(L+GL^{T}G),$$ (25)

where the components of $L_{m}$ contain the spectroscopic properties of M, namely, the linear (horizontal–vertical and 45 to 135 deg) and circular (left–right) diattenuation and retardance. Specifically, $L_{m}$ relates to the (spatial or temporal) average of the spectroscopic properties as^62^

$$L_{m}=(\begin{array}{cccc} 0 & -LD_{H} & -LD_{45} & CD\\ -LD_{H} & 0 & CB & LB_{45}\\ -LD_{45} & -CB & 0 & -LB_{H}\\ CD & -LB_{45} & LB_{H} & 0 \end{array}),$$ (26)

where LD and LB indicate linear diattenuation and retardance, respectively; CD and CB are circular diattenuation and retardance, respectively; and the sub-indexes H and 45 indicate if the linear properties are with respect to the horizontal or +45  deg axes, respectively. On the other hand, $L_{u}$ contains the necessary data to calculate their average variances ($|\Delta L_{H}{|}^{2}$, $|\Delta L_{45}{|}^{2}$, $|\Delta C{|}^{2}$) and covariances ($\Delta L_{H}\Delta L_{45}^{*}$, $\Delta L_{H}\Delta C^{*}$, $\Delta L_{45}\Delta C^{*}$).

The main assumption the differential decomposition makes is that a Mueller matrix M can be created from infinite differential matrices m. Then, for this decomposition to be applicable, each of the differential m (or L) must have physical meaning by themselves. This condition must be fulfilled to apply the differential decomposition, which can be done by verifying if the reduced coherency matrix, related to the G-symmetric component of m (or L), is positive semi-definite. The complete formulation is available in the work of Ossikovski and Devlaminck.^63^

Given the unknown nature of the optical path length inside a sample, the differential model is particularly useful for the analysis of thin samples in a transmission configuration, although its use has also been verified in the backscattering configuration.^64^

### 2.5 Classification

K-nearest neighbors classification^65^ was used to determine the usefulness of Mueller-derived properties (Δ, R, and D) for distinguishing between GM and WM. First, the dataset was balanced by randomly removing points so that each specimen and each class had the same number of samples. Then, the accuracy of the classifier was evaluated through sixfold cross-validation by leaving one specimen out on each fold. With this distinction, 83% of the data was used for training and 17% for testing.

In total, eight different instances of K-nearest neighbors (KNN) were trained: one for each wavelength ([$\Delta (\lambda_{i})$, $R(\lambda_{i})$, $D(\lambda_{i})$], i∈[1,6]), one combining specific features (SF, KNN-SF), and one containing the features at all wavelengths (KNN-All, $\left[\Delta (\lambda_{1}),R(\lambda_{1}),D(\lambda_{1}),\ldots ,\Delta (\lambda_{6}),R(\lambda_{6}),D(\lambda_{6})\right]$). The models’ average sensitivity and specificity were retrieved after sixfold cross-validation. For each fold, a confusion matrix and its respective significant parameters were obtained. The presented results were computed by averaging after the six folds. As identifying GM and WM is a binary classification problem, if we assume the “positive” class as GM and the “negative” class as WM (Table 4), sensitivity is the true GM rate (i.e., of the values detected as GM, those that are actual GM) and specificity is the true WM rate (i.e., of the values detected as WM, those that are actual WM), as indicated in Eq. (27).

$$\text{True }\mathrm{GM}\text{ Rate}=\frac{\text{True }\mathrm{GM}}{\text{True }\mathrm{GM}+\text{False }\mathrm{WM}}\;\text{True}\mathrm{WM}\text{ Rate}=\frac{\text{True }\mathrm{WM}}{\text{True }\mathrm{WM}+\text{False }\mathrm{GM}}\;\text{Accuracy}=\frac{\text{True }\mathrm{WM}+\text{True }\mathrm{WM}}{\text{True }\mathrm{WM}+\text{False }\mathrm{GM}+\text{True }\mathrm{GM}+\text{False }\mathrm{WM}}.$$ (27)

**Table 4:** Confusion matrix in a binary classification problem.

		Expected	
		GM	WM
Predicted	GM	True GM	False GM
	WM	False WM	True WM

The importance of each property for classification was analyzed through the average accuracy decrease via random permutations. This method was first introduced for random forest classifiers,^66^ but it can be applied to any model, given that only the model output is evaluated. For each property, the values are randomly shuffled so that inconsistencies between the feature’s values and the classes are introduced. If the property is important for the classifier, then its accuracy will decrease with respect to the un-shuffled dataset. We calculate the average accuracy decrease by performing the random shuffling s=10 times for each feature, fold, and model.

In addition, both forward and backward sequential floating selection (SFS; forward SFS: SFFS; backward SFS: SBFS) were implemented for KNN-All to select the three optimal features to use for KNN-SF. This method, which has been extensively documented^67^^–^^69^ and implemented for optically obtained data multiple times,^70^^–^^72^ works by choosing a subset of N features based on an optimization metric. In this case, for a dataset $X=\{x_{1},x_{2},\ldots ,x_{i}\}$ with i features, only N features are selected $X_{\text{subset}}=\{x_{1},\ldots ,x_{N}\}$, and the optimization metric is evaluated. In the next step, a new feature $x_{\mathrm{inc}}$ is included in $X_{\text{subset}}$, and another one $x_{\mathrm{exc}}$ is excluded. If $x_{\mathrm{inc}}$ provides better results than without it, $x_{\mathrm{inc}}$ is introduced in $X_{\text{subset}}$. If removing $x_{\mathrm{exc}}$ provides better results than keeping it, $x_{\mathrm{exc}}$ is removed from $X_{\text{subset}}$. This process goes on until the optimal $X_{\text{subset}}$ is reached. To avoid trying all possible combinations, once a feature is removed, it is not included again at any point in the sequential process. In SFFS, features are added until N is reached, and then, data are included or excluded without having more than N in any iteration, whereas SBFS starts with the whole dataset and removes features until N is reached, without having less than N in any iteration. Choosing SFFS or SBFS does not typically lead to the same results given that the order in which features are removed is different. In addition, depending on if N is closer to i or to 0, one method will be faster than the other. Both SFFS and SBFS were tested fold-wise for N=3 features. The idea was to obtain the three optimal features that provide the best classification without increasing the computation time regarding the single-wavelength models.

The advantage that SFS has regarding the random permutation method is that it is also not restricted to any model, and thus, the optimization metric can be any statistically relevant parameter of the data itself. In addition, SFS should be faster in these experiments than random permutations because the former evaluates metrics with fewer features (i.e., less data). However, depending on the optimization metric, other considerations might need to be evaluated, such as the type of distribution that the data have or the scale of the different features. In this case, we still opted for using the accuracy of KNN-All as the optimization metric to see if a noise-inducing method such as random permutations can provide equivalent results to a data reduction method and to evaluate if the speed increment given by SFS working with less data is worth its implementation even if it does not try all possible combinations.

## 3 Results

**Fig. 2:**
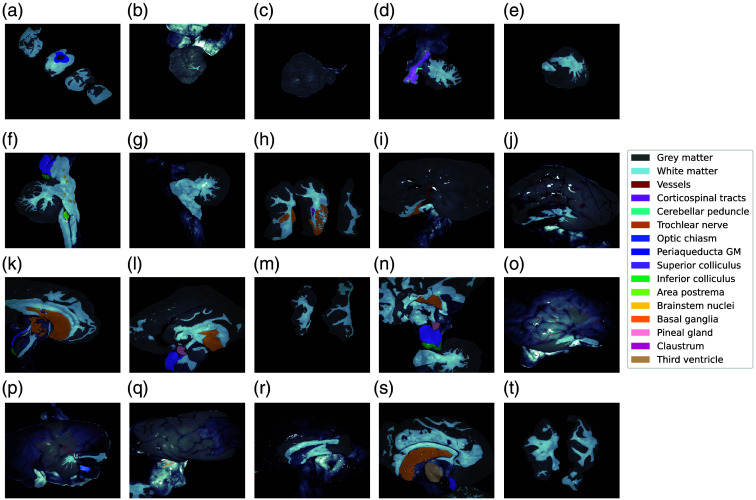
Labels identified on the 20 measurements taken of lamb brain in multiple regions: brainstem (a), lateral (b, c), and medial (e)–(g) views of cerebella, frontal basal ganglia sections (h), lateral (i, j), medial (k, l), and sectional (m) views of left hemispheres; and pineal region (n), lateral (o)–(q), medial (r, s), and sectional (t) views of right hemispheres.

The measurements from 20 different views (four macro-regions described in Table 2 and labeled as indicated in Table 3) of lamb brain were acquired according to the protocol described in Sec. 2. For each color filter, the Mueller matrices M were measured and evaluated according to their physical realizability (Sec. 3.1). In addition, the IPPs and anisotropy coefficients were retrieved (Sec. 3.1.1), and three decomposition methods (forward polar, symmetric, and differential) were applied to evaluate the resulting M (Secs. 3.1.2–3.1.4). Afterward, each image was hand-labeled by a neurosurgeon (GA author) to identify the regions introduced in Table 3, which are also depicted in Fig. 2 (Sec. 3.2). The labeled regions were then analyzed using the forward polar (or Lu–Chipman) decomposition due to its simplicity, widespread use in the literature, and prevalence as the preferred decomposition method for brain tissue analysis, facilitating comparisons with existing research. Specifically, the Lu–Chipman parameters are provided for grey–white matter distinction (Sec. 3.3) and for all labeled areas (Sec. 3.4).

### 3.1 Mueller Matrices

**Fig. 3:**
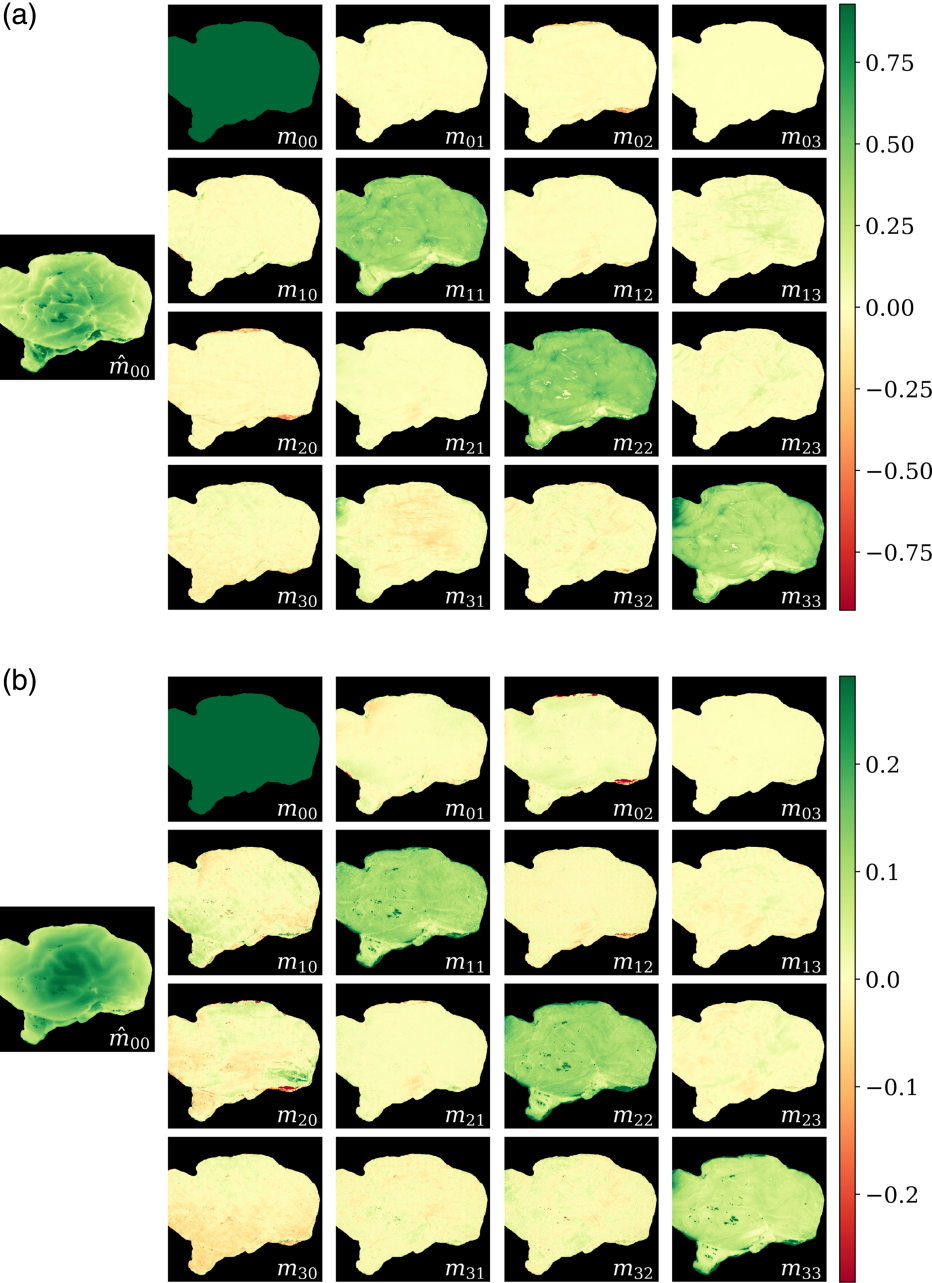
Mueller matrices of a lateral view of one left hemisphere at (a) 450 and (b) 680 nm. The non-normalized first element of the matrix, $M_{00}$, is represented to the left, whereas the rest of the coefficients, $m_{ij}$ (i,j∈[0,3]), are normalized. The colormaps were adjusted to the ±99th percentile of $M_{00}$. Positive values are showcased in green, and negative ones are depicted in red.

The measured Mueller matrices, M, with normalized components $m_{ij}$ (i,j∈[0,3]) and first (not normalized) element $M_{00}$ were almost diagonal for all samples and wavelengths, indicating that samples are mostly depolarizing. Figure 3 represents the matrix of one of the samples, at 450 and 680 nm. The finer detail observable at shorter wavelengths (450 nm) is due to the reduced blurring associated with shorter optical path lengths.

To further analyze the matrices, their physical realizability was evaluated according to the ensemble criterion. This criterion establishes two conditions for a Mueller matrix to be physically realizable, as previously stated in Sec. 2.4.1: (1) all eigenvalues of the associated Hermitian matrix, H(M) [Eq. (3)], must be non-negative, and (2) the forward and reverse passivity conditions [Eq. (4)] must be fulfilled. A tolerance of $tol=10^{-5}$ was established to fulfill conditions (1) and (2) to compensate for numerical approximations.

**Fig. 4:**
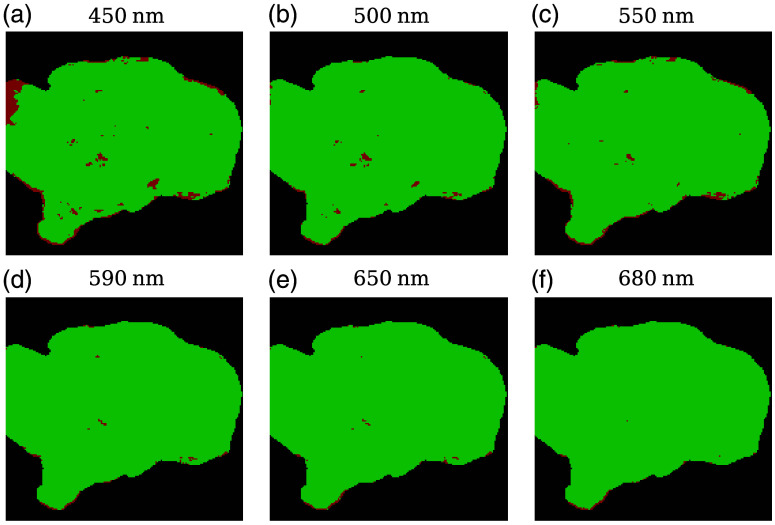
Ensemble criterion applied to one representative sample at different wavelengths: (a) 450 nm, (b) 500 nm, (c) 550 nm, (d) 590 nm, (e) 650 nm, and (f) 680 nm. Areas where the ensemble criterion is fulfilled are represented in green, and areas where the matrices are not physically realizable are in red.

The pixel-wise analysis of the ensemble criterion revealed two distinct regions within the matrices: physically realizable and non-realizable (Fig. 4). Three primary areas fail to meet the criterion: regions of specular reflection, edges of the sample where water accumulated, and areas of lower signal in samples placed over aluminum foil [Fig. 4(a)]. When measuring the initial specimens (1 and 2), reflections from the aluminum foil interfered with the samples. To mitigate this, a paper plate was used as a base for subsequent measurements. Although the images presented in this work show the entire specimens, areas where the ensemble criterion failed were excluded from the boxplots and any calculations of average polarimetric magnitudes. This effectively removed all imaging artifacts from the analysis.

#### 3.1.1 Indices of polarimetric purity indices (IPPs) and anisotropy coefficients

**Fig. 5:**
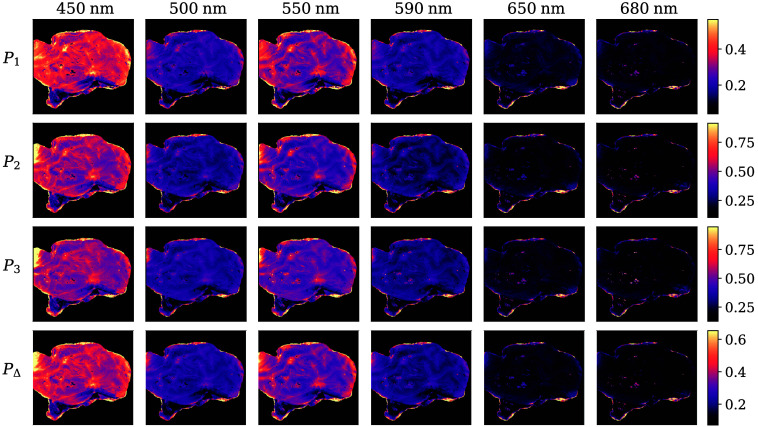
Indices $P_{1}$, $P_{2}$, and $P_{3}$ and degree of polarimetric purity $P_{\Delta }$ represented at all six wavelengths. The colormaps were adjusted to exclude all values below the 1st and over the 99th percentiles of each magnitude.

The IPPs [Eq. (5)] and the degree of polarimetric purity [Eq. (6)] were calculated for all samples. In general, the behavior is equivalent to that showcased in Fig. 5. Aside from a slight increase at 550 nm, the general tendency is that the polarimetric purity decreases as the wavelength increases. White matter structures are distinctly characterized by low polarimetric purity, indicating greater depolarization than grey matter. At longer wavelengths, virtually no polarimetric purity is retained, which is consistent with the expected depolarization increment due to the longer optical paths. Although the indices $P_{1}$, $P_{2}$, and $P_{3}$ consistently satisfy their inequality conditions, no clear relationship among them is apparent.

**Fig. 6:**
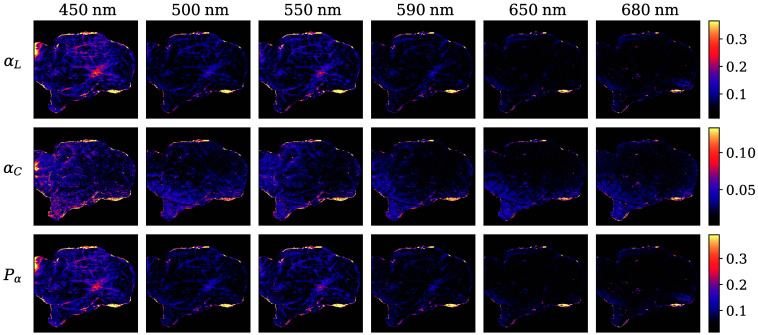
Linear ($\alpha_{L}$) and circular ($\alpha_{C}$) anisotropy coefficients and degree of anisotropy $P_{\alpha }$ represented at all six wavelengths. The colormaps were adjusted to exclude all values below the 1st and over the 99th percentiles of each magnitude.

All of the anisotropy coefficients provided very small absolute values, as required by $P_{\alpha }\leq P_{\Delta }$ (Fig. 6). Notably, linear anisotropy was almost three times the value of circular anisotropy, dominating $P_{\alpha }$ and suggesting the presence of linear retardance or diattenuation. Although some sub-cortical structures are apparent at shorter wavelengths, no significant structures were discernible above 600 nm.

#### 3.1.2 Forward polar decomposition

**Fig. 7:**
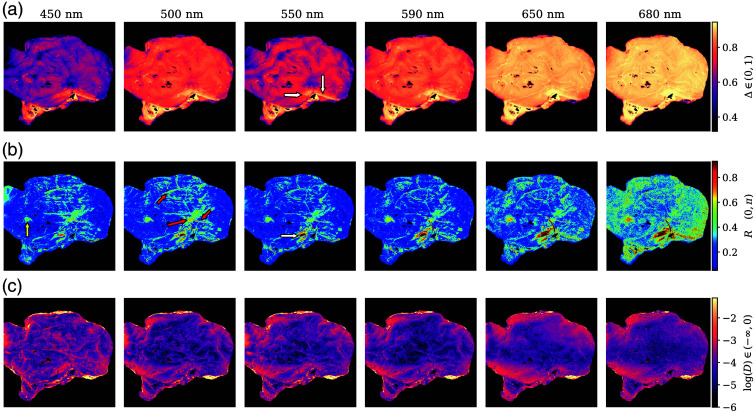
Depolarization (a), retardance (b), and diattenuation (c) of a lateral view of one left hemisphere, from 450 nm to 680 nm. The white arrows indicate white matter presence, the red ones point to some vessels, and the yellow one to sub-cortical structures. D is represented logarithmically. The data were clipped to Δ∈[0.31,0.94], R∈[0.052,0.93], and $D\in [2.5\times 10^{-3},0.33]$ for visualization purposes.

After applying the forward polar decomposition, the overall depolarization Δ, retardance R, and diattenuation D were obtained for all samples. Some of their representative images are displayed in Fig. 7 for one of the lateral views of a left brain hemisphere. For representation, the color scales are restricted to 99% of the data distribution by clipping values under 0.5% and over 99.5% (Δ∈[0.31,0.94], R∈[0.052,0.93], $D\in [2.5\times 10^{-3},0.33]$). In addition, D is depicted logarithmically for image enhancement.

Depolarization [Fig. 7(a)] provides good discrimination between GM and WM structures, with the latter having higher Δ at all wavelengths. Generally speaking, Δ increases with wavelength in all of the observed brain structures, regardless of their type. Retardance also increases slightly with wavelength [Fig. 7(b)], but the different R between matter types enhances specific structures. Blood vessels are also better detected in R out of the three parameters. The WM structures present on the hemisphere and identified through Δ are also visible in the retardance images and enhanced when the wavelengths are longer. The longer penetration of this wavelength range enhances the ability of the system to see deeper structures, as is the case with WM. However, at 680 nm, the combination of the light source used, the transmittance of the wavelength filter, the sensitivity of the camera, and the depolarization increment make it harder to discern fine structures due to the increase in noise. Low diattenuation values were observed for all samples, with D having more detail for 450 nm and being almost completely random at 680 nm. Overall D values were significantly small as tissues are not typically diattenuating. The combined effect of the higher resolution at 450 nm with its shallower penetration allows for better discrimination of superficial structures.

The behavior depicted in Fig. 7 was observed for all samples, with Δ being the main discriminant factor between WM and GM, as well as the increasing tendency for Δ and R correlating with wavelength and the overall low and non-discriminating values of D. Vessels have better visibility in the R images than in Δ images, and detail is better preserved for shorter wavelengths. Sections 3.3 and 3.4 introduce a more in-depth analysis of the results for WM, GM, and the aforementioned labeled regions.

#### 3.1.3 Symmetric decomposition

**Fig. 8:**
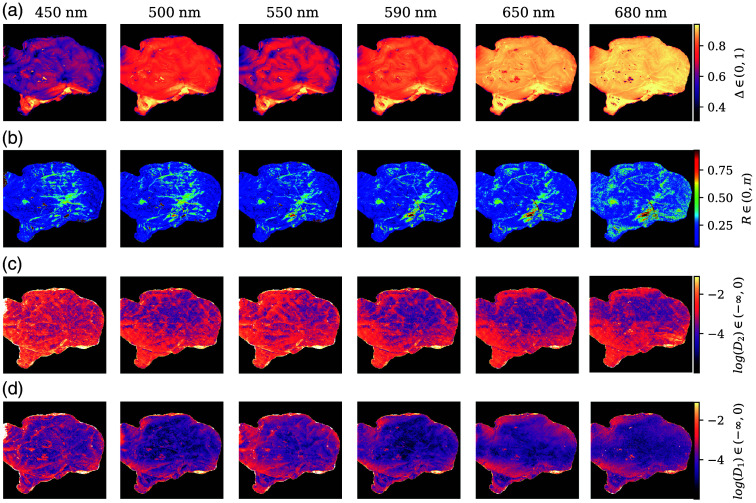
(a) Depolarization (Δ), (b) retardance (R), and (c,d) diattenuation ($D_{1,2}$) obtained with the symmetric decomposition. $D_{1,2}$ are represented logarithmically. The data were clipped to the same values used for the forward polar decomposition (Fig. 7), namely, Δ∈[0.31,0.94], R∈[0.052,0.93], and $D\in [2.5\times 10^{-3},0.33]$, for visualization and comparison purposes.

Following the procedure applied for the forward polar decomposition, the overall Δ, R, $D_{1}$, and $D_{2}$ were obtained and are represented in Fig. 8, with the same color ranges that were applied to Fig. 7.

The depolarization images [Fig. 8(a)], as derived from the diagonal depolarizer matrix, $M_{\Delta d}$, are almost identical to those provided by the Lu–Chipman decomposition. Again, there is an increasing tendency in general, with the exception of those values at 550 nm. Furthermore, Δ serves as the main discriminator parameter between GM and WM, with enhanced separability at shorter wavelengths. The overall retardance R calculated from $M_{R}={M_{R2}M_{R1}}^{T}$ [Eq. (20)] also increases slightly with wavelength and is stronger in WM structures and vessels. The main distinction between R as obtained from the Lu–Chipman and the symmetric decompositions is that the latter seems to be more resistant to noise at longer wavelengths, even with the high depolarization over 600 nm. In addition, the difference between GM and WM is less drastic than for the forward decomposition, but it is still more than twice the value for WM than for GM, and the overall increment in R is also smaller. The noise resistance of the symmetric decomposition suggests that the Lu–Chipman decomposition might overestimate the retardance values, but it does not seem to change the relative relationship between tissue types. Finally, the diattenuation obtained from $M_{D1}$ and $M_{D2}$ is also slightly different, with $D_{1}$ being more similar to the forward polar decomposition’s D, but overall diattenuation values are still negligible.

#### 3.1.4 Differential decomposition

The differential decomposition analysis of brain samples revealed no clear patterns of diattenuation or retardance, both characterized by small, non-negligible, variable values across samples and wavelengths (as shown in Table 5 for one left hemisphere). In addition, their variances are between one and three orders of magnitude greater than the values, which indicates strong depolarization. Their covariances are also small, and much smaller than the variances, which is an indicator that there is not a straightforward relationship between the polarization properties fluctuations. However, there is a 20% decrease, approximately, in the variances at 550 nm, which again supports the slight decrease in depolarization at said wavelength, as pinpointed by the other decomposition methods. These findings point toward a complex polarimetric behavior of brain tissue, dominated by depolarization effects.

**Table 5:** Wavelength-dependent linear (L), circular (C), retardance (B), and diattenuation (D) and their variances and covariances as obtained with the differential decomposition for the sample represented in Fig. 3.

	450 nm	500 nm	550 nm	590 nm	650 nm	680 nm
$LB_{H}$	0.0000	−0.0054	−0.0070	−0.108	−0.0153	0.0168
$LB_{45}$	−0.0188	−0.0309	−0.0122	−0.0062	−0.0086	0.0047
*CB*	−0.0084	0.0266	−0.0166	−0.0166	0.0202	−0.0177
$LD_{H}$	0.0025	0.0140	0.0002	0.0055	0.0138	0.0082
$LD_{45}$	−0.0201	−0.0103	−0.0132	−0.0090	0.0068	0.0082
*CD*	−0.0060	−0.0186	−0.0034	−0.0102	−0.0205	−0.0157
$|\Delta L_{H}{|}^{2}$	0.6033	1.1881	0.8964	1.2967	1.8431	1.8742
$|\Delta L_{45}{|}^{2}$	0.7293	1.3739	0.9987	1.3872	2.0271	1.9715
$|\Delta C{|}^{2}$	0.4365	0.7736	0.5527	0.8269	1.1884	1.259
$\Delta L_{H}\Delta L_{45}^{*}$	0.0166	−0.0354	0.0143	0.0067	−0.0416	0.0025
$\Delta L_{H}\Delta C^{*}$	0.0330	0.0703	−0.0040	−0.0202	0.0229	−0.0321
$\Delta L_{45}\Delta C^{*}$	0.0044	−0.0023	0.0177	0.0202	0.0061	0.0382

### 3.2 Anatomical Considerations and Rationale for Labeling

The labeling process followed the basic principles of topographical landmarks, focusing on the most recognizable structures. Two public atlases were used for comparison with our specimens.^44^^,^^45^

The lamb and human brain differ from both a functional and topographical point of view. Therefore, obviously recognizable cortical and subcortical GM structures (e.g., the basal ganglia or the periaqueductal grey) were labeled accordingly. We assigned the label of GM to those structures that were either known to be constituted entirely of GM (e.g., cortex) or showed visual/topographical features of that sort (e.g., brainstem nuclei).

Regarding the WM, the definition of precise boundaries between specific bundles was much more challenging; therefore, our segmentation was limited to the identification of the most evident nerves (optic chiasm, olfactory bulbs, etc.) or tracts, whereas we labeled all subcortical, diencephalic, cerebellar, and brainstem areas showing clearly myelinated features as generic “WM.” One important exception was made for the cortico-spinal tracts at the level of the cerebral peduncle, mainly considering the precise topographical appearance, which is also the reason that this specific structure was included in Sec. 3.4.3.

A few more structures were uncertain in nature. For example, the superior and inferior colliculi are known to be relay nuclei for the visual and auditory pathways, respectively, but they are surrounded by white matter. The same challenge applies to the third ventricle, an area rife with small grey matter nuclei and white matter bundles. In such cases, we opted to label these structures according to their macroscopic conventional name and appearance, without segregating the specific sub-components. Although this choice most likely lacks accuracy, both options of leaving the structure itself unlabeled or attempting to be more specific were not considered feasible. Interestingly, the MMI analysis still showed a relatively distinct set of features when comparing these structures to the more identifiable ones (see below).

### 3.3 Grey and White Matter

**Fig. 9:**
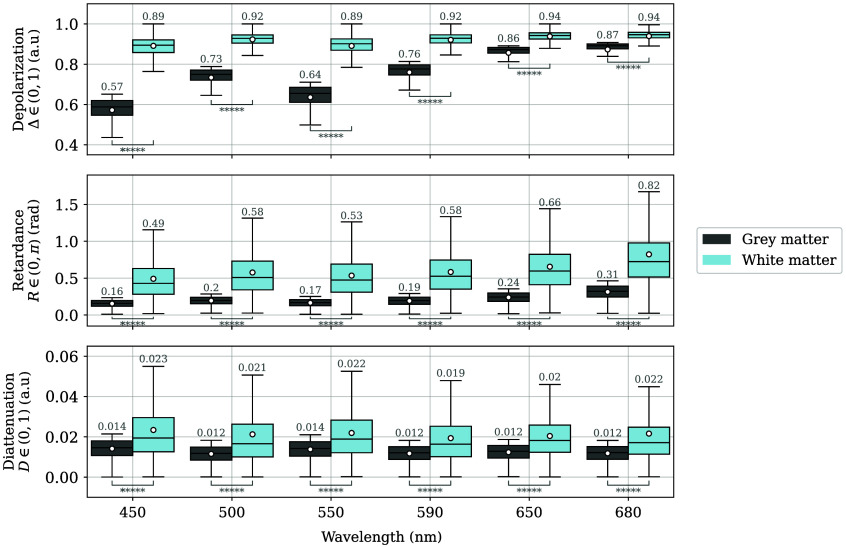
Depolarization Δ, retardance R, and diattenuation D for GM and WM, sorted by wavelength. The two-sided Mann–Whitney U test indicated p-values of p<1e−5 (*****) for all pairs GM–WM. The average of each distribution is indicated both as a white scatter point and as the number on top of each boxplot.

All of the pixels labeled as GM (1,369,395 pixels) and WM (597,686 pixels) were evaluated together in terms of depolarization, retardance, and diattenuation, regardless of the sample and brain part. The objective was to establish the capability of these polarization properties to discern between white and grey matter. Due to the uneven number of pixels in both types of tissue, we took a random sample for GM so that it contained the same number as in WM. The results are shown in Fig. 9. Among the three mentioned polarization properties, Δ exhibits the most distinct behavior. For GM, the Δ values increase with wavelength from 0.6 to almost 0.9, whereas WM values have less variability, starting just below 0.9 and reaching almost 0.95 for the longer wavelengths. The only exception is at 550 nm, where both magnitudes decrease slightly. In addition, the width of the distributions decreases for GM and WM as the wavelength increases, indicating more spatial uniformity for the red than for the blue, as previously presented in Fig. 7(a). The greatest separation between GM and WM is achieved for 450 nm, where there is no overlap between distributions.

R also increases with wavelength for GM and WM. In particular, at 680 nm, the median value for GM (0.32 rad) is twice the value at 450 nm (0.16 rad). Similarly, for WM, the value reaches 0.72 rad at 680 nm while starting at 0.42 rad at 450 nm.

As shown in Fig. 9, D has negligible values (D<0.04) at all wavelengths, especially for GM, with values that are almost half of those of WM. Further, the bulk of the distributions overlap both between GM and WM and among the different wavelengths for each type of matter.

A two-sided Mann–Whitney U test was performed to assess the overall separability of the distributions for all GM–WM pairs. All obtained p-values were p<1e−5, indicating that the three reported polarization properties have distinct distributions for each type of brain matter in the analyzed wavelength range.

Two types of KNN models were trained to distinguish between GM and WM: with single-wavelength or multiple-wavelength features. For both types, the neighbor number was established by analyzing the cross-validated accuracy for the test dataset between $K_{n}=1$ and $K_{n}=100$. After $K_{n}=50$, the accuracy increased only an additional 0.05% for the single-wavelength models, whereas the all-wavelength one peaked at $K_{n}=8$, and the specific-features one at $K_{n}=48$, so those were the values chosen for each model type. The average accuracy for the train and test datasets is shown in Table 6. For all models, true GM and WM rates are close to the accuracy, both for training and for testing, indicating that no model is biased toward one class or the other.

**Table 6:** Average accuracy, true GM rate, and true WM rate after sixfold cross-validation for the train and test datasets for each of the eight KNN models trained.

	Accuracy (train)	Accuracy (test)	True GM (train)	True WM (train)	True GM (test)	True WM (test)
450 nm	89.10 ± 0.72	88.3 ± 9.5	88.98 ± 0.73	89.22 ± 0.71	88.7 ± 7.8	88.3 ± 9.5
500 nm	88.52 ± 0.82	85.9 ± 7.9	88.44 ± 0.82	88.59 ± 0.83	86.3 ± 6.4	85.9 ± 7.9
550 nm	88.84 ± 0.57	87.8 ± 6.8	88.68 ± 0.58	88.98 ± 0.56	88.0 ± 5.7	87.8 ± 6.8
590 nm	87.25 ± 0.78	86.5 ± 6.6	87.14 ± 0.78	87.36 ± 0.78	86.6 ± 5.5	86.5 ± 6.6
650 nm	83.47 ± 0.89	82.5 ± 7.7	83.22 ± 0.94	83.71 ± 0.86	82.4 ± 6.6	82.5 ± 7.7
680 nm	79.9 ± 1.4	79.0 ± 8.5	79.7 ± 1.4	80.2 ± 1.4	78.8 ± 7.4	79.0 ± 8.5
KNN-All	97.25 ± 0.49	95.3 ± 6.8	97.23 ± 0.49	97.27 ± 0.49	95.5 ± 6.1	95.3 ± 6.8
KNN-SF	95.57 ± 0.65	94.1± 8.4	95.57 ± 0.64	95.57 ± 0.65	94.6 ± 7.6	94.1 ± 8.6

KNN-All stands for the models trained with Δ, R, and D at all six wavelengths, whereas KNN-SF was trained specifically with Δ(450,500,and 550  nm). Results are shown in percentages and presented with two significant figures.

**Fig. 10:**
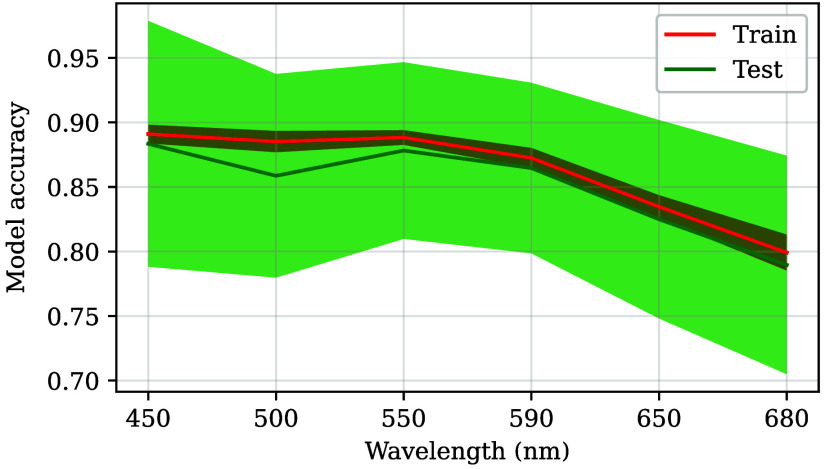
Average accuracy (solid lines) and its standard deviation (shaded areas) after sixfold cross-validation for the train (red) and test (green) datasets. The data depicted in this image correspond to single-wavelength models.

Below 590 nm, KNN correctly identifies the type of tissue (on average) in over 85% of the training dataset cases for the single-wavelength models (Fig. 10). Between 590 and 650 nm, that number is reduced to around 81%, and it keeps decreasing to 77% for 680 nm. The standard deviation due to the test folds changes between 12% and 18%, with the smallest one at 590 nm, whereas the training dataset results are better at 550 nm. In addition, there is not a big difference between the average training and test results (∼2%), and the test dataset completely overlaps with the training set due to the former’s greater standard deviation.

**Fig. 11:**
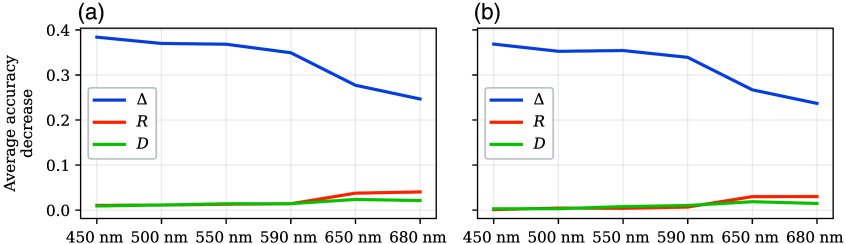
Average accuracy decrease through feature permutation of depolarization (blue), retardance (orange), and diattenuation (green) for the train (a) and test (b) datasets for the single-wavelength models. In this case, each model uses only the features (Δ, R, and D) for each wavelength at a time, without any additional information. The standard deviation is included as shaded areas around each line, but at the scale of the graphs, it is not visible.

Looking at the feature importance of the three polarization properties (Fig. 11), we see that the main discriminating one is Δ, which is the one that reduces accuracy the most through random removal, and its values are between three and four times those of R and D. This correlates with the distributions depicted in Fig. 9, given that Δ boxplots do not overlap at all below 650 nm and R and D have some amount of overlap at all wavelengths. Only from 650 nm and above does the importance of Δ start to decrease and the classifiers look at R and D as well, but their contribution is not enough to keep the accuracy constant.

Two additional models were trained: one that contained all features (KNN-All) and another composed specifically of Δ(λ=450,500,and 550  nm) (KNN-SF). The rationale behind adding said models was to see if multiple-wavelength data can outperform using only one λ. In the case of KNN-SF, we selected N=3 features for the training times to be comparable with the single-wavelength models. Their results are compared with the single-wavelength models in Table 6. Overall, the accuracy of KNN-All outperformed all other models, reaching a 95% accuracy for the test dataset, which is an indicator that multi-wavelength setups provide more clinically relevant information than single-wavelength ones.

**Fig. 12:**
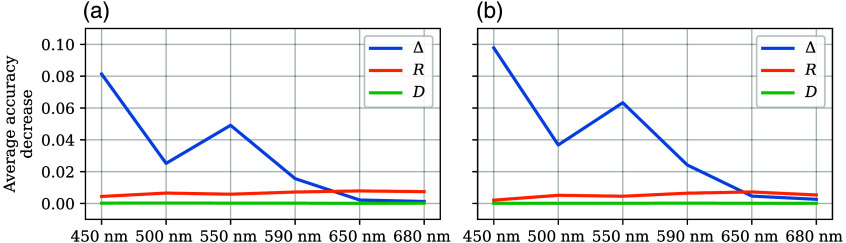
Average accuracy decrease through feature permutation of depolarization (blue), retardance (orange), and diattenuation (green) for the train (a) and test (b) datasets for the all-wavelengths model. In this case, the model uses all available features (Δ, R, and D) at all wavelengths. The standard deviation is included as shaded areas around each line, but at the scale of the graphs, it is not visible.

To choose the parameters for KNN-SF, we evaluated the average accuracy decrease through random permutations with KNN-ALL (Fig. 12). Similar to the single-wavelength models, depolarization is the principal parameter that contributes to the accuracy; however, its impact is four times less than for the single-wavelength models. This indicates that having multispectral information makes the classifiers more robust to noisy features. The importance of the wavelengths still decreases with the wavelength increment, similar to what was observed for the single-wavelength models, both for the test and train datasets. An exception to this trend is that the results at 550 nm seem to contribute more to the accuracy than those at 500 nm.

SFFS and SBFS were also introduced in this step to see if the best-selected features by SFS were comparable to the random permutation models. In all folds, SFFS and SBFS provided the same results. Specifically, Δ(450  nm) was the best feature for all folds, corresponding to what random permutations indicate for KNN-All. The second-best feature was Δ(500  nm) for the first five folds, whereas for the last one (specimen 1 for testing), the second-best feature was Δ(550  nm). Finally, the third best feature selected varied: Δ(550  nm) for the first four folds, and Δ(590  nm) for the last two. On average, the best features were the ones chosen for KNN-SF, namely, Δ(λ=450,500,and 550  nm), but interestingly, doing random permutations or SFS interchanges the second and third best features, which, if we had chosen N=2, would give rise to two different datasets for classification. However, evaluating 10 random permutations on KNN-All with sixfold cross-validation implied training for almost 2 h per fold because each fold fits 10 models that consider hundreds of thousands of data points. On the contrary, SFFS starts with only one feature and builds up to N, whereas SBFS starts with all of them (18) and removes features up until N. This gave training times of ∼3  min for SFFS and 30 mins for SBFS (per fold), which makes SFS much more manageable even if not all possible data combinations are tested with these methods.

The choice of optimized features leads to over a 6% increase in accuracy as obtained with KNN-SF with respect to all single-wavelength models and only 1% less than with KNN-All (Table 6), without increasing the computation time, which was less than a minute per fold. Overall, selecting key multispectral features appears to be the best way to obtain good classification results.

### 3.4 Analysis by Brain Region

The results provided in this subsection are grouped according to the areas described in Table 2. To make a complete description of each anatomic region of the brain, the labeled regions identified within them (Fig. 2) are also discussed in this section. The pineal region and basal ganglia images [see Figs. 2(n) and 2(h), respectively] are contained within the hemisphere’s discussion (Sec. 3.4.1) due to the labeling difficulties discussed in Sec. 3.2. GM and WM are still included in this section, specifically referring to each anatomical region. The only exception is the pineal region, where the GM and WM labeling was excluded due to it being a mixture of hemispherical and cerebellar GM and WM.

#### 3.4.1 Brain hemisphere, optic chiasm, basal ganglia, and claustrum

**Fig. 13:**
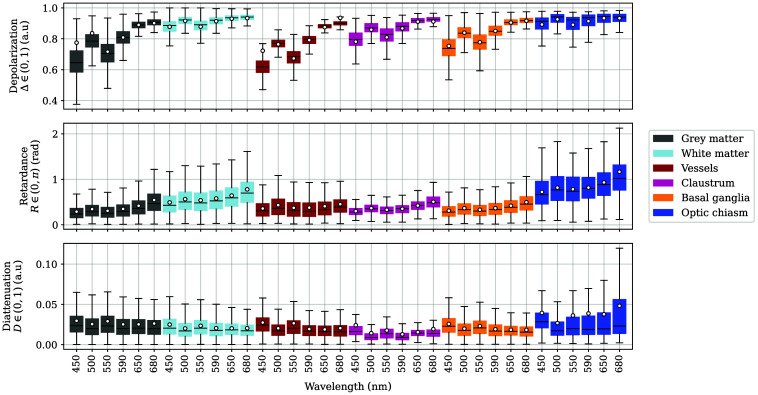
Depolarization Δ, retardance R, and diattenuation D for the labeled regions on the hemispheres. The first two colors correspond to GM and WM, respectively. The remaining data are sorted by ascending the 75th percentile of retardance at 680 nm from left to right. The average of each distribution is indicated as a white scatter point. Outliers are not shown.

A total of 11 of the 20 captured regions belonged to brain hemispheres and adjacent areas [Figs. 2(i)–2(m) and 2(o)–2(t)]. Between the left and right hemispheres, a total of 10 unique areas were identified. In the lateral views, GM, WM, and blood vessels were easily detectable, along with some areas of the basal ganglia and optic chiasm. On the hemisphere sections, only cortical GM and subcortical WM were visible, but additional basal ganglia and claustrum data from the frontal basal ganglia image [Fig. 2(h)] are included here, too. Both Δ and R increase with increasing wavelength for all tissue types (Fig. 13). As indicated previously, Δ values for GM and WM form almost a continuum of values of depolarization with increasing wavelength. Vessels have polarimetric optical properties that overlap GM and WM significantly, which is consistent with their positioning over both types of hemispheric matter and their reduced thickness. The remaining regions also have polarimetric values ranging between those of GM and WM. As expected, the Δ values of the basal ganglia are closer to GM’s values, whereas those of the optic chiasm lie nearer to WM’s values.

Hemispheric R shows values between 0 rad and π rad, but the bulk of R data is between 0 and 1 rad, specifically for lower wavelengths. In this case, the width of the inter-quartile ranges increases for longer wavelengths for most labels, especially for those of the claustrum and GM, which exhibit a retardance almost two times stronger at 680 nm than its value at 450 nm. Structures such as vessels, the basal ganglia, and the claustrum have retardance values closer to those of GM, whereas the optic chiasm retardance is even greater than those of WM.

As mentioned, diattenuation for hemispheric tissue is very low, with most values being under 0.05. However, we detected a slight decrease of D with increasing wavelength for most tissue types, but the overlap between categories makes D the least discriminant feature of the three.

#### 3.4.2 Cerebellum

**Fig. 14:**
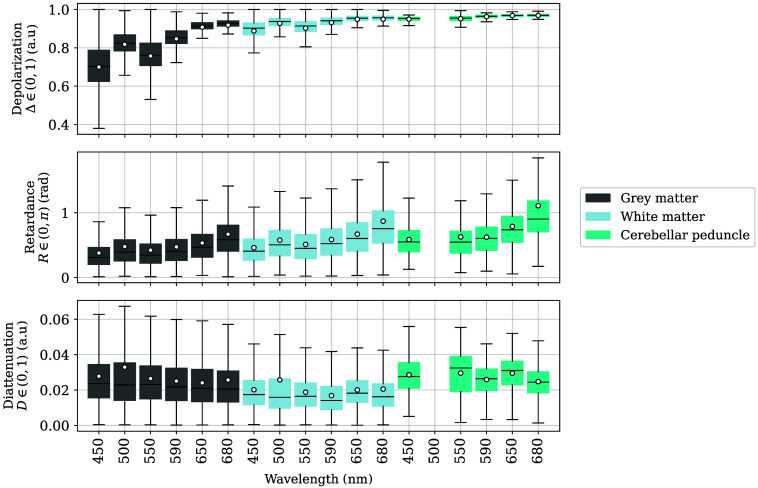
Depolarization Δ, retardance R, and diattenuation D for the labeled regions on the cerebella. The first two colors correspond to GM and WM, respectively. The remaining data are sorted by ascending the 75th percentile of retardance at 680 nm from left to right. The data of the cerebellar peduncle at 500 nm were removed due to sample movement artifacts. The average of each distribution is indicated as a white scatter point. Outliers are not shown.

Complete Mueller measurements of six cerebellar areas were captured and labeled with six distinct tags [Figs. 2(b)–2(g)]. Some of the lateral and medial views were taken with the cerebellum still connected to the brainstem [Figs. 2(d), 2(f), and 2(g)]; therefore, some images also included labeled sections from the area postrema, brainstem nuclei, corticospinal tracts, and cerebellar peduncle. Only cerebellar GM, WM, and the cerebellar peduncle are included in this section; the remaining areas in the cerebellar images are considered in Sec. 3.4.3. The data of the cerebellar peduncle at 500 nm were not considered in this analysis due to unintentional movement artifacts, which prevented the co-registration of Mueller images. Specifically (Fig. 14), GM and WM have values of Δ, R, and D in the same ranges and with the same behavior as that already discussed for the hemispheres.

The cerebellar peduncle has Δ values superior to those of WM, even though it is a WM structure itself, and its boxplots have a smaller interquartile range than GM or WM, possibly due to the reduced number of labeled pixels by comparison. Retardance-wise, all three types of matter provided very similar results, with the cerebellar peduncle again having values slightly above GM and WM. Finally, the calculated diattenuation was also low in cerebellar matter (D<0.04). No significant behavior was observed for any of the labels in the D images either.

**Fig. 15:**
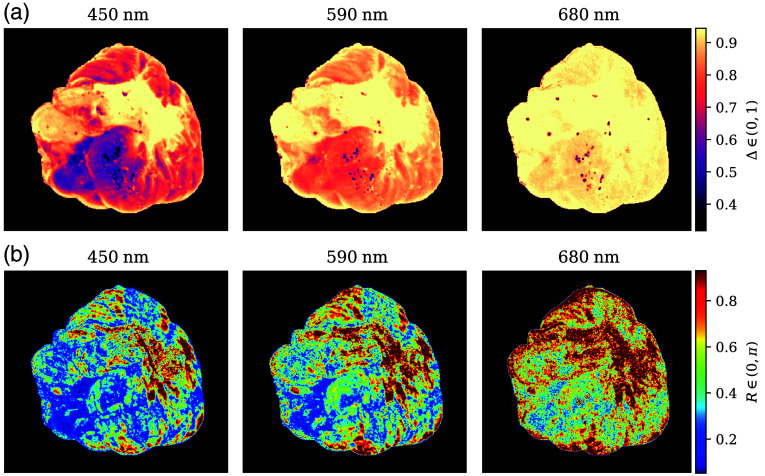
Depolarization (a) and retardance (b) of a medial view of one cerebellar hemisphere, at 450, 590, and 680 nm. The color representation is chosen to coincide with Fig. 7.

Images of the cerebellar regions behaved as discussed in Sec. 3 with some exceptions on R. We expected the linear retardance to enhance WM structures as it did in the brain hemispheres (Fig. 7), and at those locations where Δ indicated WM presence, R values were also significantly high. However, WM structures were not as well defined in cerebellar R values as for the hemispheric WM (Fig. 15). In particular at 430 nm, only the biggest stem of the arbor vitae is highlighted by R, whereas its structure is perfectly delineated by Δ. The contrary happens as the wavelength increases, with a better delineation of retarding tissue, but at the same time, the increased amount of depolarization results in noisier images.

#### 3.4.3 Brainstem

As mentioned in Sec. 3.4.2, some of these images [Figs. 2(d) and 2(f)] contained brainstem-related structures, such as the trochlear nerve, area postrema, brainstem nuclei, pineal gland, and superior and inferior colliculi. The latter two were also identified in multiple medial views [Figs. 2(k), 2(l), and 2(n)], as well as the third ventricle, present in Fig. 2(s). Due to the aforementioned labeling difficulties, only GM and WM from the brainstem section image [Fig. 2(a)] are discussed in this section.

**Fig. 16:**
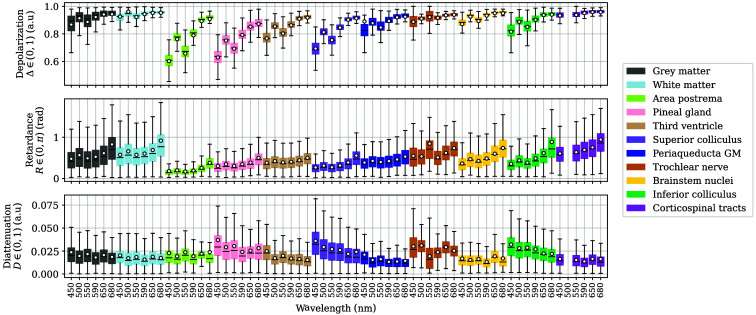
Depolarization Δ, retardance R, and diattenuation D for the labels in the brainstem, pineal region, and frontal basal ganglia areas. The first two colors correspond to GM and WM, respectively. The remaining data are sorted by ascending the 75th percentile of retardance at 680 nm from left to right. The data of the corticospinal tracts at 500 nm were removed due to sample movement artifacts. The average of each distribution is indicated as a white scatter point. Outliers are not shown.

The section image [Fig. 2(a)] was specifically interesting due to its complex combination of GM and WM, which is clearly reflected in the fact that GM and WM are more similar to each other than for the hemispheres or cerebellum in all three magnitudes (Fig. 16). Periacqueductal GM was independently labeled due to its localization and structure, and its depolarization and diattenuation are highly similar to regular brainstem GM, with only its retardance being slightly less than for GM. Specifically, the Δ values of the area postrema, pineal gland, third ventricle, and superior colliculus are closer to GM’s values, but their lower limits are inferior, whereas the inferior colliculus matches GM in Δ. By contrast, the Δ values of the trochlear nerve, brainstem nuclei, and corticospinal tracts lie nearer to WM values. Retardance-wise, the periaqueductal GM lies in between both WM structures (trochlear nerve, corticospinal tracts) and most GM structures (area postrema, pineal gland, third ventricle, superior colliculus). However, brainstem GM and WM have wider interquartile ranges than the other brainstem structures, which were easier to delimit when labeling. Finally, D does continue the aforementioned tendency for the other tissue types, with very low values and little to no differentiation between tissues.

### 3.5 Optical Axis of Retardance and White Matter Tracts

**Fig. 17:**
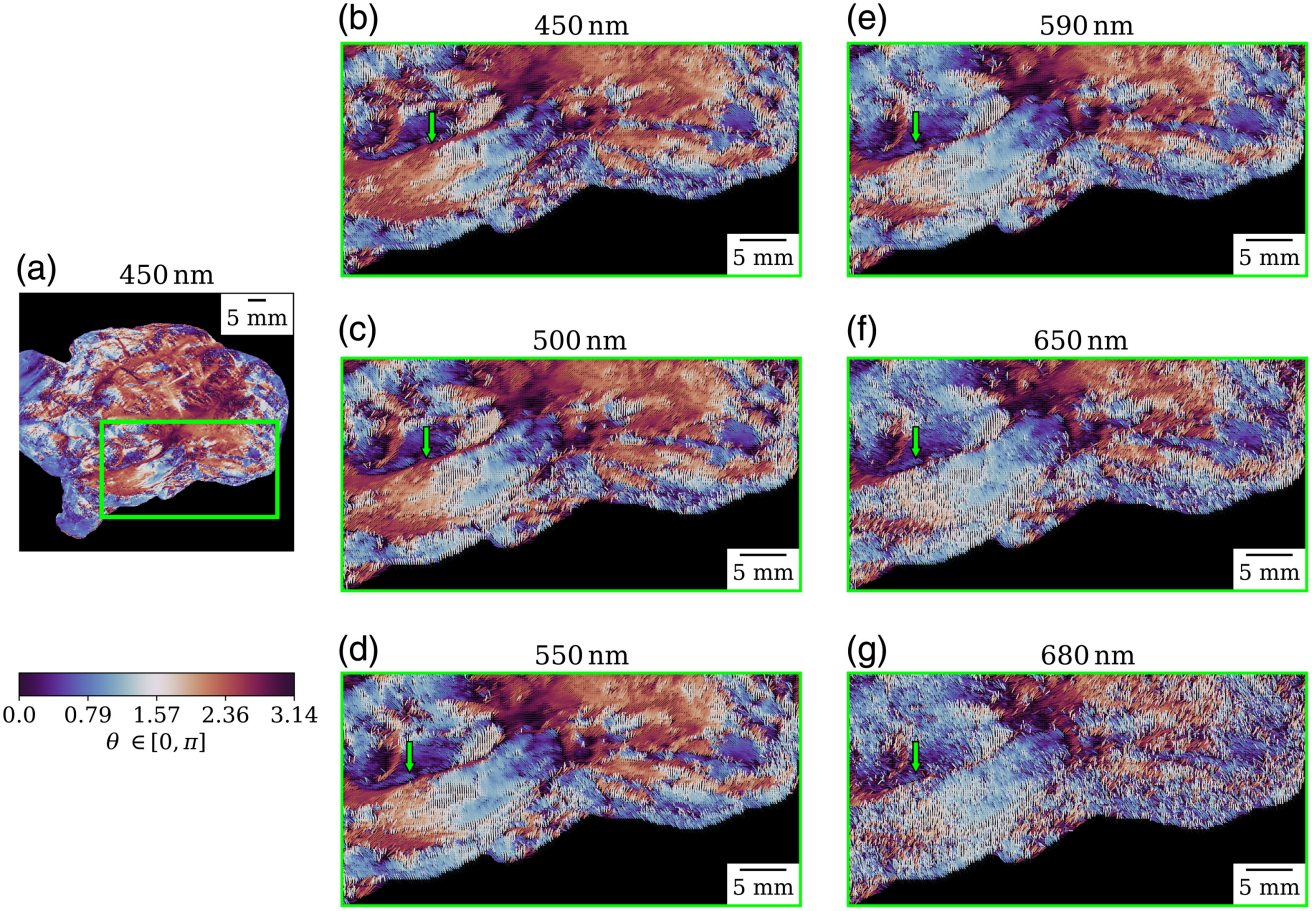
Orientation of the optical axis of linear retardance (θ) for the left hemisphere depicted in Fig. 7: (a) reference image at 450 nm, (b)–(g) optical axis orientation at the six wavelengths for the area marked by the green square in panel (a). The green arrow marks show that the frontier between GM and WM moves from right to left with the different wavelengths. All images share the same color scale.

The optical axis of linear retardance (θ) was calculated for all Mueller matrices. A representative image is depicted in Fig. 17. Discerning between GM and WM is not trivial by looking at one single wavelength because there are slight variations throughout the whole specimen that make delineation harder than using Δ or R. In Fig. 17(a), an area containing GM and WM was marked as a region of interest to evaluate at all wavelengths. We observed that the frontier between GM and WM (as viewed in Δ) moves slightly when the wavelength increases, revealing more detail of the WM tracts at longer wavelengths. An example of this phenomenon is indicated by the green arrow in Fig. 17. As for the other parameters, surface structures are highlighted for sub-550 nm wavelengths, especially the interface among some major gyri, some vessels, and overall regions with similar properties. Beyond 550 nm, the signal starts to deteriorate due to a decrease in the signal-to-noise ratio, combined with the blurring effects produced by longer optical path lengths at greater wavelengths, making it harder to discern specific characteristics.

## 4 Discussion

This work focuses on reporting the polarization properties of healthy brain tissue when obtained through multispectral MMI in animal samples.

The choice of whole, fresh brain specimens was based on two main factors. First, we assumed that the comparison between our results and the findings on fixed tissue reported in the literature remains valid. This assumption is supported by recent evidence demonstrating the effect that fixation has in Mueller-derived parameters of brain tissue, with Gros et al.^36^ finding a slight depolarization increase for GM and a 27% to 28% retardance decrease in GM and WM. Second, we aimed to gather a realistic dataset in a non-invasive manner before applying this technology to an *in vivo* scenario. Although formalin effects have been quantified as mentioned above and can be taken into account in the discussion, we hypothesized that the texture and the mechanical properties of fresh tissue without fixing agents are closer to those seen during a potential *in vivo* acquisition. For the purpose of acquiring a rich and heterogeneous dataset, views from multiple specimen sections (comparable to those of a neurosurgical procedure) and different angles were considered in this work.

The proposed system can be easily replicated due to the wide availability of the optical elements. Reflectance systems often imply the need for an oblique angle detector, which in our setup was ∼7  deg. Although single backscattering from reciprocal media yields reciprocal Mueller matrices with only 10 independent parameters, the introduction of an oblique angle can break these symmetries. Additional factors such as multiple scattering, non-uniform samples, and instrumental noise and accuracy can further contribute to the loss of symmetry. However, an inspection of our obtained Mueller matrices revealed predominantly diagonal (depolarizing) matrices, suggesting that the off-diagonal elements, although influenced by the aforementioned phenomena, remain relatively small. Nevertheless, it is important to consider that depolarization tends to be the most accurately derived parameter in such scenarios.

The measured Mueller matrices of brain samples had an almost-diagonal composition; thus, the polarimetric indices and all decomposition methods point to the elevated depolarization power of the samples, a characteristic frequently encountered in biological tissues. Analysis of the polarimetric indices indicates significant differences between grey and white matter, with the latter exhibiting a near-complete loss of polarimetric purity, consistent with its denser composition and lower absorption. The progressive loss of polarimetric purity as the wavelength increases is an expected behavior due to the increment in multiple scattering. Regarding the anisotropic properties, the samples predominantly displayed linear anisotropy, with minimal to no circular anisotropy detected. The absence of significant circular anisotropy suggests a limited net contribution of chiral structures to the overall polarization properties and a polarimetric predominance of structural features with preferential orientations, such as white matter tracts. The absence of significant circular anisotropy was further corroborated by the components derived from the forward polar decomposition. In all cases, linear retardance was one order of magnitude higher than circular retardance. Specifically, linear retardance (both respect to the horizontal and 45 deg axes) had averages ranging from 0.2 rad at 450 nm to 1.0 rad at 680 nm, approximately, and the average circular retardance stayed under 0.2 at all wavelengths. In the case of the overall low diattenuation, circular diattenuation was approximately half or less of the value of its linear counterparts.

The multispectral analysis setting revealed that the main discriminating factor between GM and WM was depolarization (Δ) for all tissue types, with WM having values close to 1 for all wavelengths and GM values increasing steadily from 450 to 680 nm. This previously reported GM–WM distinction through Δ^24^^,^^25^^,^^36^ was corroborated by our analysis of this phenomenon at six wavelengths, with the differences between matter types being higher for shorter wavelengths. A possible explanation for having more distinguishable distributions at 450 nm could be that the dominating scattering of WM over GM is also enhanced by the anisotropy of the tissue, i.e., the fiber-like organization of WM, as suggested by Alali et al.^23^ Therefore, the Δ increment of GM and WM at longer wavelengths could be caused by the scattering and birefringence behavior of each tissue type evolving at different rates, thus giving different separability of the Δ distributions. The absorption and scattering coefficients of GM and WM have been extensively analyzed by Shapey et al.^73^ In their study, GM and WM had similar absorption profiles that generally decrease between 400 and 600 nm, with the only exception to that trend being the hemoglobin peaks around 550 nm. However, the scattering coefficient of GM decreased, whereas, for WM, it initially increased and then decreased in the same wavelength range, which could be the origin of the different rate of change that we observed in the depolarization. Another possibility is that, as absorption is lower at 680 nm than at 450 nm,^2^^,^^73^ there would be two regimes of Δ: the highly scattering, highly absorbent regime at shorter wavelengths and the low scattering, low absorbent regime at longer wavelengths. The latter would therefore showcase longer optical path lengths that result in more multiple-scattering events that would randomize the resulting Δ distributions, even if, overall, the scattering probability decreases.^2^^,^^73^ At the same time, shallower penetration lengths will make polarization preservation easier for shorter wavelengths. All of these factors (absorption, scattering, and birefringence) might therefore play a role in showing more separability between GM and WM for shorter than for longer wavelengths, and further research would provide more insight into this field to model the exact causes of depolarization in brain tissue.

In all distributions (Figs. 9, 13, 14, and 16), there was a dip in depolarization at 550 nm. Multiple studies^24^^–^^26^ attribute this change in Δ to the absorption peaks of the hemoglobin, which is known to be one of the main chromophores in tissue.^2^ There are other additional chromophores in the brain, such as eumelanin and lipofuscin, which do not present any characteristic absorption or scattering signal at this wavelength.^74^ The sensitivity of Δ to the hemoglobin is also supported by our experiments by the fact that the vessels are areas where the Δ change with the wavelength at 550 nm is most visible, followed by GM structures (Fig. 13). Specifically, the median value of Δ at 550 nm for GM is 9% lower than the tendency marked by the values at 500 and 590 nm, whereas, for WM, the dip represents a 2% decrease. Combining the fall in Δ with the knowledge that the cerebral blood volume (CBV) is (3±0.4)  ml/100  g in GM and (1.7±0.4)  ml/100  g in WM^75^ and that temporal variation in detected CBV could correspond to variation in perfusion, multispectral MMI could be a highly sensitive method to potentially determine not only the blood volume but also the oxygenation level in the brain through the addition of hemoglobin-specific wavelength filters. Nonetheless, it is worth noting that most multi-wavelength studies that focused on calculating tissue absorption considered very few chromophores, mostly due to the limited number of available reference spectra. Therefore, more in-depth studies of brain chromophores in the visible range are encouraged to have a complete description of their influence in Δ.

Still, depolarization was the most helpful parameter for classifying GM and WM. We tested eight K-nearest neighbors classification models based on different single-wavelength or multi-wavelength features. All of them focused more on Δ than on any other parameter, especially at shorter wavelengths. In addition, using only depolarization data as selected by SFS or random permutations was almost as good as using all available features for classification, as long as multi-wavelength information was kept for training. This indicates that multispectral/hyperspectral setups can provide better results than single-wavelength ones even with traditional classification methods. It is worth noting that Δ was the only feature apparently influenced by tissue absorption, which again highlights the necessity for multimodal systems that provide complementary structural and compositional information.

In a similar experiment to what is explored in this work, Bonaventura et al.^33^ analyzed in depth the change in retardance with the wavelength (405 to 632 nm) for three brain regions (corticospinal tracts, cerebellum, and optic chiasm). It is worth noting that, although for our analysis the retardance increased with the wavelength regardless of tissue type, thickness, or structure, their analysis showcased different results for each tissue type. Specifically, their results for the cerebellum differ from ours in that retardance decreases on average. There are multiple reasons that these differences might occur. In the aforementioned study, fixed ferret brains were used, whereas this work considers fresh lamb specimens. Aside from the anatomical differences that come with using different animals, fixed brain tissue can showcase almost a third less retardance than fresh tissue,^36^ as previously indicated, which could be the cause of the retardance differences between both publications. However, the effect of formalin in terms of the wavelength has yet to be explored. In addition, their setup consists of a modified microscope, which should provide higher spatial resolution than our wide-field lens. This implies that, although their microscope can resolve small spatial features, our system would be averaging some GM/WM structures if they are too small and interlaced. For the optic chiasm, their experiments report an increase in the retardance, which is slightly different between the left and right lobes, with average values around 0.3 rad. In our case, the optic chiasm was visible in a side view of one right hemisphere (Fig. 2), and its average values increased from 0.8 to over 1 rad (Fig. 13). Even though both our and their results show an increase in retardance, the average differences and their variances make comparisons difficult when considering all of the mentioned factors that make both experiments differ. Finally, for the corticospinal tracts, Bonaventura et al. reported no absolute increase between 405 and 442 nm, with an average of ∼0.7  rad, which is within the interquartile range of the values that we calculated at 450 nm for the same tissue type (Fig. 16). Thus, an interesting line of future research would be to measure the same samples and reproduce the experiments to see how both setups compare and complement each other, as well as to potentially find a bridge between the micrometric scale given by a high-resolution polarimetric microscope and the wide-field real-time applicability of a macroscopic setup.

When considering the bulk of WM and GM measured in all samples and sample types, all parameters had distinct distributions at all wavelengths. However, those distributions are less separated when focusing on individual labels (Figs. 13, 14, and 16). There are many considerations to evaluate for this comparison that could be playing a role in the separability, such as the number of pixels contemplated on each label, the fiber orientations, and the local chemical or anatomical composition. However, the reduced separability is still an indicator of how the polarimetric optical properties change according to the difference between GM and WM types. For example, the optic chiasm and the trochlear nerve both fall into the WM category. Still, their different structure affects specifically their retardance (Figs. 13 and 16), with the optic chiasm having a higher average R at all wavelengths, suggesting a more anisotropically organized structure than the trochlear nerve. This finding resembles that of diffusion tensor tractography.^76^ The same rationale could be applied to all labels, with R being a marker of tissue anisotropy. Nonetheless, the longer the wavelength is, the less polarized light returns to the sensor, which indicates that retardance values could be a mixture of multiple local features and not single GM or WM structures because only the upper-most tissue layers preserve the initial polarization. The results at longer wavelengths are especially affected by the calibration error obtained when retrieving the PSA and PSG matrices, which for our system were, on average, 6.3% and, specifically, 5.6% at 650 nm and 4.5% at 680 nm, respectively. Although our findings align well with the existing literature and anatomical expectations, the observed polarimetric purity below 0.2 for wavelengths over 600 nm combined with the calibration error suggests potential inaccuracies in the decomposition-derived parameters at these wavelengths. This phenomenon could explain the observed decrease in classifier accuracy above 600 nm, despite the increased importance of retardance and diattenuation for classification performance. Further research on this topic could help determine the real penetration depth of polarized light and the depth range that preserves the polarization properties.

In general, the size of the structure to be observed should be considered when choosing a wavelength. The shorter penetration of the 450 nm light, combined with the higher anisotropic scattering signal of WM, makes it the ideal wavelength range to visualize small surface structures such as the thinner branches of the cerebellar’s arbor vitae [Fig. 15(a)]. GM mostly absorbs light at these wavelengths,^73^ which is consistent with our results given that there is no significant R or D and the Δ is at its lowest (Fig. 9), further enhancing the GM–WM contrast. This could also explain why, at shorter wavelengths, only the thickest parts of cerebellar WM are viewed as localized spots of retarding matter, as the interlaced GM–WM architecture of the cerebellum, significantly smaller than the human one, hinders the R separability. The longer path length at 680 nm causes light to pass through more of the cerebellar WM, thus outlining its retardance better. This phenomenon represents a fundamental characteristic of multispectral MMI, given that there is a trade-off between detail (Δ, short wavelengths, superficial) and structural information (R, long wavelengths, sub-superficial). The size of the different features also affects the separability of the distributions. It is known that the penetration depth of light at 450 nm for human brains is between 0.1 and 0.5 mm and between 1 and 2 mm for 650 nm and that GM can have twice as much as WM inside those ranges.^77^^,^^78^ In addition, light penetration is not downward-facing, but it spreads horizontally, too. Therefore, even considering animal samples, we can assume that features in the sub-mm range are going to be blurred with the neighboring and underlying tissue, which will affect the separability of all distributions, especially those with a dimmer signal (R and D) and at the longest wavelengths.

The orientation of the optical axis of retardance (θ) has also been addressed by different research groups and reported to be a non-invasive option to visualize WM tracts.^24^^–^^27^^,^^33^^,^^41^ Our multispectral analysis revealed a progression in the orientation of WM tracts and their differentiation with GM, in which each wavelength plays a role in the pseudo-tomographic assessment of θ. The anatomical explanation of this phenomenon resides in the different orientations of the WM tracts in the subcortical space as the association, projection, and commissural fibers are often merging, mixing, and/or running in different directions.^76^ The observed orientation changes with the increasing wavelength could be due to not only the observation of deeper layers inside the tissue but also the previously mentioned blurring due to adjacent structures. In a recent work in which Mueller imaging was combined with structured light, the authors reported the same effect^32^ of the delineation of GM and WM being improved with increasing the spatial frequency attributed in this case to the enhanced penetration. Bonaventura et al.^33^ also proposed that longer wavelengths are better for observing the micro-structure due to the increased penetration depth. The same authors expanded on this topic in their recent article^41^ in which, for shorter wavelengths, only superficial features were visible, but the longer ones provide averaged values over thicker tissue sections. Those experiments are consistent with our results and the enhanced spatial resolution and less noisy data that we obtained for shorter wavelengths.

Diattenuation did not provide significant information regarding any of the analyzed brain structures, at any of the wavelengths. However, an emerging area of research^79^^,^^80^ focuses on D as a marker for tissue homogeneity, fiber compactness, fiber size, and myelin sheath thickness. It has also been recently shown that D can be an indicator of fiber inclination.^41^ All of these experiments were conducted with polarization-sensitive microscopes, so it is possible that our reflectance macroscopic setup is structurally or optically inadequate to retrieve the type of detail needed to decouple the already dim diattenuation from the heavily depolarizing environment of bulk samples. Nonetheless, further research is needed to evaluate if shorter wavelengths can be used to retrieve D as a useful marker in neuroimaging.

## 5 Conclusion

As Mueller matrix imaging gains importance in clinical settings, there is a growing need for studies to establish the polarimetric behavior of all types of tissue in multiple environments. To address this, we applied multispectral Mueller matrix imaging to animal brain specimens, aiming to document and describe the polarimetric properties of healthy brain tissue. Our primary focus involved imaging, labeling, and determining depolarization, retardance, and diattenuation at six different wavelengths and using them for tissue classification.

This experiment highlights the critical role of the wavelength dependency of the polarization properties, given that high-resolution depolarization imaging can be achieved at short wavelengths, whereas longer ones are better for the analysis of tissue anisotropy through retardance. This also means that the change in penetration length with the wavelength, given by the optical properties of the tissue, allows for the visualization of structures and their orientation at different depths inside the tissue. In the brain, both depolarization and retardance play a major role in the differentiation of WM and GM. This effect was proven by training multiple classifiers and observing how much the results change or improve by considering specific wavelengths or their combination. Overall, multi-wavelength models performed better than single-wavelength ones. The orientation of the observed fibrous-like structures also changes slightly with depth; therefore, the choice of wavelength should be carefully considered, especially if the application at hand aims to image surface or sub-surface phenomena.

## Data Availability

The dataset presented in this article is publicly available in Zenodo at https://doi.org/10.5281/zenodo.11127947.
